# The Inflammasome–miR Axis in Alzheimer’s Disease and Chronic Pain: Molecular Mechanisms and Therapeutic Opportunities

**DOI:** 10.14336/AD.2025.0353

**Published:** 2026-05-21

**Authors:** Botond Gaál, Roland Takács, Csaba Matta, Krisztián Juhász, Béla Fülesdi, Zoltán Szekanecz, Szilvia Benkő, László Ducza

**Affiliations:** ^1^Department of Anatomy, Histology and Embryology, Faculty of Medicine, University of Debrecen, Hungary, Nagyerdei krt. 98, H-4032 Debrecen, Hungary.; ^2^Department of Anesthesiology and Intensive Care, University of Debrecen Medical and Health Science Center Nagyerdei krt. 98, H-4032 Debrecen, Hungary.; ^3^Institute of Internal Medicine, Department of Rheumatology, Faculty of Medicine, University of Debrecen, Debrecen, Hungary.; ^4^Laboratory of Inflammation-Physiology, Department of Physiology, Faculty of Medicine, University of Debrecen, Debrecen, Hungary.

**Keywords:** Alzheimer’s disease, Chronic pain, Neuroinflammation, Inflammasome, MicroRNA, Spinal cord, Glia

## Abstract

Alzheimer’s disease (AD) is a progressive neurodegenerative disorder characterized by cognitive decline, synaptic dysfunction, and chronic neuroinflammation. Mounting evidence suggests that inflammasome activation plays a pivotal role in the onset and progression of AD by promoting neuronal damage, Tau pathology, and amyloid-β (Aβ) accumulation. Among the various inflammasome types expressed in the central nervous system (CNS), NLRP3 has received particular attention due to its strong association with both AD and pain-related neuroinflammation. Chronic pain, frequently observed in older adults and individuals with dementia, shares overlapping inflammatory mechanisms with AD, including glial activation and cytokine dysregulation. The inflammasome–microRNA (miR) axis has recently emerged as a key regulatory pathway modulating these neuroinflammatory responses. Specific inflammation associated miRs, such as miR 22, miR 34a, miR 146a, miR 155, and miR 223, influence innate immune signaling and critically affect both neuronal homeostasis and pain sensitization. Emerging evidence also implicates dysfunction of the locus coeruleus–noradrenergic (LC–NE) system—an early target of AD pathology—in amplifying neuroinflammation and pain sensitivity, partly through interactions with dysregulated miRs. While previous studies have addressed the roles of inflamma-miRs in AD or chronic pain individually, this review uniquely examines their interconnected roles—highlighting how dysregulated miR expression and inflammasome activation may converge to drive persistent neuroinflammation across both conditions. By elucidating shared molecular pathways, we propose that targeting the inflammasome–miR axis may offer dual therapeutic potential: slowing AD progression while addressing pain-related neural dysfunction. As the prevalence of AD rises, such integrated insights are essential for the development of more precise, mechanism-based interventions.

## 1. Introduction

Chronic pain, as defined by the International Association for the Study of Pain (IASP), is "an unpleasant sensory and emotional experience associated with, or resembling that associated with, actual or potential tissue damage" lasting longer than three months [1,2]. It is a leading cause of disability and mortality worldwide, significantly affecting quality of life and imposing a substantial socioeconomic burden [3]. The prevalence of chronic pain varies due to methodological differences, cultural factors, and healthcare disparities. Additionally, socioeconomic and political determinants, such as national health expenditures and governance quality, influence pain-related outcomes and access to care [4–6].

Unlike acute pain, which serves as a protective physiological response, chronic pain is a maladaptive condition sustained by complex neurobiological mechanisms, including peripheral and central sensitization, altered neuron-glial interactions, and dysregulated neuroimmune signaling [7–12]. Beyond biological factors, psychosocial components such as anxiety, depression, catastrophizing, and self-efficacy also modulate pain perception and treatment response [13–15]. Given its complexity, chronic pain management requires an interdisciplinary, multimodal approach, with growing evidence supporting integrative rehabilitation strategies over monotherapies [16].

Alzheimer’s disease (AD), the most common form of dementia, is a progressive neurodegenerative disorder characterized by memory loss, cognitive decline, and synaptic dysfunction, ultimately impairing daily functioning. Chronic pain frequently coexists with AD, with studies estimating that up to 46% of AD patients experience persistent pain [17–21]. However, due to cognitive impairment and communication barriers, the true prevalence of chronic pain in AD may be underestimated [22,23]. Functional MRI and pain reflex assessments suggest that AD patients not only perceive pain but may experience altered pain processing, potentially due to disease-related neurodegenerative changes [24]. Notably, different dementia subtypes exhibit distinct pain responses; for example, frontopolar dementia is associated with increased pain thresholds, whereas vascular dementia patients display pain responses similar to healthy individuals [25–27]. Increasing evidence suggests that neurodegeneration may contribute to exaggerated pain sensation in some forms of dementia [28].

Emerging research indicates that chronic pain may exacerbate neurodegenerative processes in regions of the brain implicated in AD. Several of these areas are involved in sensory integration, emotional regulation, and higher-order cognitive functions [29, 30]. Neuroimaging studies in chronic pain patients have revealed structural brain alterations, particularly reductions in gray matter volume, that mirror those observed in AD [31]. These changes have been consistently reported in the entorhinal cortex, anterior cingulate cortex, amygdala, parahippocampal gyrus, insula, and thalamus, hippocampus and prefrontal cortex—regions critically involved in memory, emotion, and executive function [32-35].

Chronic pain, like AD, is associated with structural brain alterations and dysregulation of key neurotransmitter systems, including dopamine, serotonin, and norepinephrine (NE)—all of which are implicated in AD pathology [36, 37]. These neurotransmitters are essential for regulating mood, cognition, and pain perception. In AD, amyloid-β (Aβ) contributes to these disturbances through its synaptotoxic effects, even at nanomolar concentrations. Persistent overproduction of Aβ at dendrites or axons leads to a reduction in synaptic number and plasticity [38]. Additionally, Tau—a predominantly presynaptic protein—can modulate Aβ’s effects at the postsynaptic site. Hyperphosphorylated Tau (p-Tau) accumulates in dendritic spines, disrupting synaptic trafficking and further impairing neuronal communication [39].

Notably, alterations in the noradrenergic (NE) system have been associated with structural and functional changes in the locus coeruleus (LC)—the brain’s primary source of NE and one of the earliest regions affected in AD. Preclinical studies indicate that chronic pain can impair NE synthesis and turnover within the LC, potentially exacerbating cognitive decline and emotional dysregulation [40, 41]. The LC is particularly vulnerable due to its anatomical proximity to the fourth ventricle, where the blood–brain barrier (BBB) exhibits increased permeability. This makes it especially susceptible to early Tau deposition and peripheral inflammatory signals [42]. Reduced NE levels in the LC can further compromise BBB integrity by downregulating tight junction proteins [43]. Disruption of the LC–NE system may, in turn, potentiate microglia-mediated neuroinflammatory responses, promoting the accumulation of Aβ and Tau. This cascade accelerates neurodegeneration and contributes to cognitive impairment. Thus, LC–NE dysfunction may represent a critical point of convergence between chronic pain and AD-related neurodegenerative processes [44].

Neuroinflammation is increasingly recognized as a central feature of AD and its associated comorbidities, suggesting common underlying mechanisms. Sustained activation of microglia and astrocytes promotes chronic inflammatory signaling, contributing to synaptic dysfunction and progressive neuronal loss [45, 46]. Supporting this, genome-wide association studies (GWAS) [47, 48], have identified numerous immune-related genes linked to AD risk, while single-cell transcriptomic analyses have uncovered disease-associated microglia (DAMs) that appear to play key roles in neurodegeneration [49].

Importantly, AD is a heterogeneous disorder characterized by variability in Aβ and Tau pathology, genetic susceptibility, and clinical presentation. These complexities, along with inconsistencies in temporal relationships among clinical symptoms, pathology, and biomarkers, underscore the challenges in establishing universal diagnostic criteria and effective therapeutic strategies.

Among the critical mediators of this inflammatory milieu are the inflammasomes—intracellular protein complexes that sense cellular stress and trigger immune responses. In parallel, dysregulated microRNAs (miRs) have been implicated in both AD [50] and chronic pain [51], yet their potential convergence has not been thoroughly explored. Notably, aberrant miR expression may impair LC–NE signaling, thereby enhancing nociceptive sensitivity and accelerating cognitive decline [52].

In this narrative review, we examine current evidence linking inflammasome activity and miR regulation in the context of AD and chronic pain, drawing primarily from peer-reviewed studies published in the past decade and identified through focused PubMed searches. We propose that the interplay between inflammasomes and miRs—here referred to as the inflammasome–miR axis—may represent a shared regulatory pathway driving persistent neuroinflammation and disease progression in both conditions.

## 2. Autoinflammation and inflammasome activation

Autoinflammatory conditions are closely associated with dysfunctions in the innate immune system [53]. The innate immune system is characterized by its broad, non-specific response to pathogens, functioning as the body’s first line of defense. This system employs both cellular mechanisms (such as phagocytes) and humoral components (such as cytokines) to combat infections and maintain homeostasis [53, 54].

Immune cells activated during innate immune responses include phagocytes (macrophages and neutrophils), dendritic cells, mast cells, basophils, eosinophils, natural killer cells and innate lymphoid cells [55]. Additionally, other cell types, such as epithelial and endothelial cells, are also induced to express molecules recognizing damage-associated molecular patterns (DAMPs) and pathogen-associated molecular patterns (PAMPs) and are classed as ‘innate responders’ [54, 56, 57].

Within the cytokine superfamilies, interleukin-1 (IL-1) family, tumor necrosis factor (TNF) superfamily members, IL-6 and the type I interferons are particularly implicated in innate immune responses [58-60]. Several molecular systems, including Toll-like receptors (TLRs), NOD-like receptors (NLRs), the caspase recruitment domain (CARD) receptor family, proteins of the complement system, cytoplasmic DNA-sensing molecules and inflammatory multimolecular complexes such as inflammasomes, have evolved to permit diverse recognition, activation and effector function within innate immunity [54].

Inflammasomes are large molecular-weight multiprotein complexes in the cytoplasm that assemble when specialized pattern recognition receptors (PRRs, sensors) detect threatening stimuli including a wide range of PAMPs and DAMPs [61, 62]. The innate immune system relies on a variety of PRRs, such as TLRs, RIG-I-like receptors (RLRs), absent in melanoma 2 (AIM2)-like receptors (ALRs), NLRs, and cyclic GMP-AMP synthase (cGAS)/STING. Another family of sensors, the ALRs, contains pyrin domain and HIN domain (PYHIN) proteins and AIM2 [63-66]. In humans, there are 14 members of NLRPs, namely NLRP1–NLR14. Under physiological conditions, NLRs maintain an auto-inhibited conformation that is released when they detect DAMPs/PAMPs. This activation leads to the assembly and activation of inflammasomes. The N-terminal pyrins (PYDs) of NLRs bind to and initiate the oligomerization of the adaptor protein termed apoptosis-associated speck-like protein with a caspase recruitment domain or CARD (ASC). It is important to note that the ASC contains both a CARD and a PYD domain. Consequently, through homotypic interactions such as CARD-CARD or PYD-PYD, ASC proteins form complexes with the PYD or CARD domains of NLRs [61-64].

In canonical inflammasomes, ASCs initiate the assembly of inactive caspase-1 zymogens through CARD-CARD interactions, leading to their polymerization and proximity-induced self-cleavage, resulting in active caspase-1. The latter process involves the conversion of precursor IL-1β and IL-18 into their mature forms, as well as the generation of N-terminal fragments of the gasdermin-D (GSDMD) protein, a key pyroptosis-executing pore-forming protein. The generation of extensive pores within the cellular membrane compromises its structural integrity, ultimately resulting in pyroptotic cell death [63-66].

## 3. CNS inflammasomes: pivotal players in the pathogenesis of AD and chronic pain

There is an increasing focus directed towards the innate inflammatory processes that occur specifically within the brain and spinal cord, collectively termed "neuroinflammation." Neuroinflammation can manifest in various contexts, including disease states, physical injuries, infections, or psychological stress. Each of these scenarios influences the nature and intensity of the inflammatory response [67, 68].

Inflammasome activation is a well-recognized key phenomenon in AD patients [69]. Most likely, it may actively drive the progression of AD, since systemic inflammation has been found to be a risk factor for the disease and appears before the onset of cognitive decline. Concomitantly, the inflammasome-driven mediators such as IL-1β or IL-18 are also released in response to tissue injury or inflammation, contributing to peripheral and/or central sensitization by sensitizing nociceptors and promoting neuron-glia crosstalk, perpetuating chronic pain [70-72].

**Table 1.** Brain NLRP1 Inhibitors Used in Preclinical Models of Neurological disorders.

Pharmacological compound	Animal / cell culture	Experimental model	Main mechanism	Reference
Intravenous immunoglobulin	Primary cortical neuron cell culture (C57BL/6J mice) C57BL/6J mice	Oxygen-glucose deprivation or stimulated ischemia-reperfusion Middle cerebral artery occlusion (Ischemic stroke model)	↓ NLRP1, NLRP3 ↓ ASC, XIAP, caspase-1, caspase-11 ↓ IL-1β, IL-18	Fann et al. 2013 [212].
Sinomenime (Morphinane alkaloid)	Sprague-Dawley rats	Pentylenetetrazole kindling chronic epilepsy model in hippocampus	↓ Bax, caspase-3 ↑ Bcl-2 ↓ NLRP1, ASC, caspase-1 ↑Cognitive function ↓IL-1β, IL-18, IL-6, TNF-α	Gao et al. 2018 [213].
Ginsenoside Rg1 (triterpene sapogenin)	Primary hippocampal neuron cell culture (postnatal Sprague-Dawley rats) APP/PS1 mice APP/PS1 mice	H_2_O_2_ induced neuronal cell damage Alzheimer’s disease model Alzheimer’s disease model	↓ Neuronal apoptosis ↓ NLRP1, ASC, caspase-1 ↓ IL-1β, IL-18 ↓ Caspase-3 ↓ NOX2, p47phox ↓Aβ, APP, BACE, Tau ↓ROS, NOX2, p22phox, p47phox ↑PSD95 ↑Cognitive function, spatial learning ↓Neuronal damage, Aβ ↓NLRP1 ↓p AMPK/AMPK, Beclin1, LC3II/LC3 I ↑p mTOR/mTOR	Xu et al. 2019 [214]. Zhang et al. 2021 [215]. Li et al. 2023 [216].
Resveratrol (3,5,4′-trihydroxy-*trans*-stilbene)	HTR-8/SVneo trophoblast cell culture (human) Swiss mice	H_2_O_2_-induced oxidative stress damage model Streptozotocin injection-induced sporadic AD model	↓ IL-1β, caspase-1, NLRP1, ↓ LC3, Beclin-1 ↓ ROS ↑Improved memory ↓Neuroinflammation	Li et al. 2020 [217]. Fonseca et al. 2023 [218].
Curcumin (polifenol, diarylhepanoid)	Fetal rat cerebral cortical neuron cell culture Sprague-Dawley rats	Oxygen-glucose deprivation Middle cerebral artery occlusion (Ischemic stroke model)	↓ NLRP1 caspase-1, GSDMD, ↓ IL-1β, IL-6, TNF-α ↓ iNOS ↓ p38 MAPK ↑ Neuroprotection	Huang et al.2021 [219].
Sarsasapogenin (Steroidal sapogenin)	Sprague-Dawley rats	Streptozotocin-induced diabetes	↓ PAR-1 ↓ NLRP1, IL-1β, IL-18, IL-6, TNF-α ↑ Cognitive function	Kong et al. 2021 [220].
Schisandrin (tannin)	SH-SY5Y neuroblastoma cell culture (human) APP/PS1 mice	Aβ_1-42_ stimulation	↓ Aβ, NLRP1, ACS, caspase-1, IL-1β, IL-18, ↓ Bax, Caspase 3 ↑ Bcl-2 ↑ Memory, learning	Li et al. 2021 [221].
Shaoyao Gancao Tang (made of P. lactiflora and *G.* uralensis at 1:1 ratio)	Aβ-GFP SH-SY5Y neuroblastoma cell culture (human) 3×Tg mice	Medium from interferon-γ-activated HMC3 microglia Streptozotocin-induced AD model	↓ NLRP1, NLRP3 ↓ ROS, iNOS, ↓ IL-1β, IL-6, TNF-α, caspase 1 ↑ Neurite outgrowth ↑ Working and spatial memory ↓ Aβ, Tau, NLRP1, NLRP3	Chiu et al. 2021 [222].
Parthenolide (sesquiterpene lactone, germacranolide)	BV2-microglia cell culture (mice) HT22-hippocampal neuron cell culture (mice) C57BL/6J mice	LPS-induced BV2 cells Oxygen glucose deprivation/re-oxygenation Traumatic brain injury model (controlled cortical impact)	↓IL-1β, IL-6, TNF-α ↑IL-4, IL-10 ↓COX2, iNOS ↓STAT3/NF-κB ↓NLRP1, NLRP3, NLRC4 ↓Bax ↑Bcl-2 ↓ Brain edema, neuronal apoptosis ↑ Memory, learning	Ding et al. 2022 [223].

↓ decrease ↑ increase **Aβ:** Amyloid beta; **AD:** Alzheimer’s disease; **AMPK:** 5′ AMP-activated protein kinase; **APP/PS1:** Amyloid Precursor Protein-Presenilin 1; **ASC:** Apoptosis-associated speck-like protein containing a CARD; **BACE:** Beta-secretase 1; **Bad:** BCL2 associated agonist of cell death; **Bax:** Bcl-2-Associated X Protein; **Bcl2:** B-cell lymphoma 2; **COX2:** Cyclooxygenase-2; **GSDMD:** Gasdermin D; **iNOS:** Inducible nitric oxide synthase; **IL:** Interleukin; **LPS:** Lipolysaccharide; **MAPK:** Mitogen-activated protein kinase; **mTOR:** Mammalian target of rapamycin; **NF-κB:** Nuclear factor kappa- B**; NLRC:** NOD-like receptor family CARD-containing 4 protein; **NLRP:** NOD-like receptor protein; **NOX2:** NADPH oxidase 2; **PAR-1:** Proteinase-activated receptor 1; **PSD95:** Postsynaptic density protein 95; **ROS:** Reactive oxygen species; **STAT3:** Signal transducer and activator of transcription 3; **XIAP:** X-linked inhibitor of apoptosis protein

The expression of inflammasomal proteins has been extensively studied, with key members such as nucleotide-binding domain leucine-rich repeat-containing protein 1 (NLRP1), NLRP2, AIM2, NOD-like receptor family CARD domain-containing protein 4 (NLRC4), and NLRP3 playing crucial roles in neuroinflammation within the CNS. These inflammasomes are implicated in a wide range of neurological disorders, including chronic pain, ischemic stroke, traumatic brain and spinal cord injuries, neurodegenerative diseases, epilepsy, and various brain infections [72-75]. To provide a comprehensive and well-structured overview, we have compiled Tables 1–3, which summarize key CNS inflammasome inhibitors and the associated signaling pathways affected in these conditions. Furthermore, Supplementary Tables 1-2 provide a concise summary of key studies implicating NLRP3 activation in AD and various pain conditions.

While certain inflammasomes like NLRP3 have known miR regulators in neuropathological conditions such as AD, more detailed on Table 4, there is currently no direct evidence of miR-mediated control of NLRP2 or NLRC4 in CNS models. Likewise, although miR-9a-5p has been shown to directly target NLRP1 and attenuate its downstream pro-inflammatory signaling in ischemic stroke models—reducing levels of cleaved caspase-1, IL-1β, and IL-18 in rat model of middle cerebral artery occlusion (MCAO) and oxygen-glucose (OGD)-exposed neuronal cells—the role of NLRP1-regulating miRs in AD or chronic pain remains unexplored [76].

Additionally, emerging preclinical research underscores the potential of miRs to modulate AIM2-driven neuroinflammation. For instance, M2 microglial exosomes enriched with miR-672-5p suppress the AIM2 inflammasome and neuronal pyroptosis in spinal cord injury models [77]. In ischemic brain injury, miR-485 has been shown to target AIM2, while maternally expressed gene 3 (MEG3) exacerbates inflammation by sponging miR-485 [78]. Collectively, these studies suggest that miR–inflammasome regulatory networks may represent promising therapeutic targets, although their relevance to AD pathology remains insufficiently characterized.

### 3.1 NLRP1 inflammasome

NLRP1 (also known as NALP1) was the first molecular platform of the NLR family identified in ischemic murine brain and rat spinal cord injuries [79, 80], being highly expressed in the CNS predominantly in neurons and microglial cells [81-83]. In contrast to NLRP3, NLRP1 possesses a C-terminal extension that includes a CARD domain, which has been documented to engage directly with procaspase-1, thereby eliminating the necessity for ASC. Notable genetic variations exist between murine and human NLRP1, indicating potential functional differences that have evolved over time. While the human NLRP1 is encoded by a single gene, mice have three genes that can exhibit up to six different haplotypes, designated *NLRP1 a, b, c, d, e, and f* [84].

#### 3.1.1 NLRP1 inflammasome in *in vivo* models of AD

Multiple reports have highlighted the involvement of inflammasomes in AD-related neuroinflammation, with particular emphasis on the NLRP1 inflammasome. Studies have shown that the expression of NLRP1 is altered in the brains of double transgenic mice (APP/PS1) expressing amyloid precursor protein (APPswe) and presenilin-1 (PS1dE9), suggesting a role for NLRP1 in the pathophysiology of AD. Recent studies also suggest that inhibiting NLRP1 can alleviate dysfunctions related to autophagy mediated by 5′-monophosphate-activated protein kinase (AMPK) and the mammalian target of rapamycin (mTOR), and improve Aβ clearance in APP/PS1 model [85].

Recent hypotheses suggest that neuronal loss may play a more critical role in the progression of AD than the accumulation of Aβ or the activation of microglia. Notably, research has demonstrated that inhibiting caspase-1 in aged AD mice can improve cognitive function without significantly altering inflammation or Aβ accumulation in hippocampal microglia [86].

In streptozotocin (STZ) induced rat AD model, increased expression of inflammasome components, including NLRC4, ASC, and IL-1β, was observed in the hippocampus. However, no discrepancies were found in the expression of other inflammasome components such as NLRP1, NLRP3, AIM2, and IL-18 [87]. These discrepancies highlight the complexity of inflammasome regulation, which may vary across species, brain regions, and cell types, as well as in response to experimental conditions [88].

**Table 2.** Preclinical Brain Inhibitors of NLRP2, AIM2, and NLRC4 in Neurological Disorders.

Pharmacological compound	Animal / cell culture	Model	Main mechanism	Reference
Brilliant blue G (P2X7 receptor antagonist) Probenecid (Urocosuric agent, pannexin 1 inhibitor)	Primary astrocyte cell culture (human)	ATP-stimulation	↓ NLRP2, caspase 1 ↓ IL-1β, IL-18 ↓ NLRP2, caspase 1 ↓ IL-1β, IL-18	Minkiewicz et al. 2013 [102]
Exenatide/exendin 4 (GLP-1 receptor agonist)	Primary astrocyte cell culture (human) 5×FAD mice	Aβ_1-42_-induced oxidative stress and inflammation	↓ NLRP2, caspase 1 ↓ GFAP ↓ ROS ↓ IL-1β, IL-18, TNF-α ↓ NLRP2, ↓ IL-1β, TNF-α ↓ GFAP ↑ Cognitive function	Zhang et al. 2022 [105]
Obovatol (biphenyl lignan)	IRC mice Human APP mutant Tg2576 mice LPS-primed bone marrow-derived macrophages (C57BL/6 mice) C57BL/6 mice	Aβ_1-42_ intracerebroventricular infusion model of Alzheimer’s disease Alzheimer’s disease model Combined activation of NLRP3, AIM2, NLRC4 (nigericin, dsDNA-*Listeria monocytogenes* infection flagellin) MSU induced acute gout model	↑ Cognitive function ↓ GFAP ↓ NF-kB ↓ i-NOS, COX2 ↑ Spatial memory ↓ Aβ ↓ BACE ↓ GFAP ↓ AIM2, NLRP3 ↓ ASC, caspase 1, (pro)-IL-1β ↓ ROS ↓ IL-1β	Choi et al. 2012 [224] Kim et al. 2019 [225]
Methylene blue organic chloride salt, phenothiazinium (Oxidation-reduction agent)	APP/PS1 mice LPS-primed bone marrow-derived macrophages (C57BL/6 mice) LPS-primed monocyte-like THP-1 cells (human)	Alzheimer’s disease model AIM2 activation model: dsDNA transfection and *Listeria monocytogenes* infection NLRC4 activation model :flagellin or inoculated with *Salmonella* typhimurium Combined AIM2 and NLRC4 activation model, nigericin, NLRP3 activation)	↑ Cognitive function ↓ Aβ ↓ ROS ↓ Caspase 1, IL-1β ↓ Phagocytosis ↓ AIM2 ↓ NLRC4 ↓ Caspase 1, IL-1β ↓ NLRP3, NLRC4, AIM2	Paban et al. 2014 [226] Ahn et al, 2017 [227]
Indomethacin (NSAID)	Sprague-Dawley rats	Streptozotocin-induced Alzheimer- like condition	↓ NLRC4, NLRP3 ↓ IL-1β, IL-18, ASC ↓ Caspase 1, p-Tau ↑ Cognitive function	Karkhah et al. 2021 [228]

↓ decrease ↑ increase **Aβ:** Amyloid beta; **AIM2:** Absent in melanoma 2; **APP/PS1:** Amyloid Precursor Protein-Presenilin 1; **ASC:** Apoptosis-associated speck-like protein containing a CARD; **BACE:** Beta-secretase 1; **COX-2:** Cyclooxygenase-2; **dsDNA:** Double-stranded DNA; **5XFAD:** 5 familial AD mutations; **GFAP:** Glial fibrillary acidic protein; **GLP-1:** Glucagon-like peptide-1; **iNOS:** Inducible nitric oxide synthase; **IL:** Interleukin; **LPS:** Lipolysaccharide; **MSU:** Monosodium urate; **NF-κB:** Nuclear factor kappa-B; **NLRC:** NOD-like receptor family CARD-containing 4 protein; **NLRP:** NOD-like receptor protein; **NSAID:** Nonsteroidal anti-inflammatory drugs; **ROS:** Reactive oxygen species

#### 3.1.2 NLRP1 inflammasome in *in vitro* and human models of AD

Aβ has been shown to elevate NLRP1 expression in rodent brain, leading to the activation of caspase-1 signaling. This signaling cascade is crucial for triggering neuronal pyroptosis and the release of pro-inflammatory cytokines, indicating that the NLRP1/caspase-1 pathway contributes significantly to the neurotoxic effects of Aβ [89].

Supporting these findings, Kaushal et al. [90] demonstrated that the assembly of NLRP1 in serum deprived human primary neuron cultures leads to sequential activation of caspase-1, followed by caspase-6. This cascade was found to elevate the Aβ_42_ ratio further implicating the NLRP1/caspase-1/caspase-6 signaling pathway in AD. Importantly, in the brains of individuals with AD, NLRP1 expression was found to be 25 to 30 times higher than in healthy controls, with elevated levels of active caspase-6 identified within abnormal neuritic plaques, neurofibrillary tangles, and nerve threads in both sporadic and familial types of AD [91-93].

**Table 3.** NLRP3 Inhibitors in Preclinical and Clinical Studies for Neurological Disorders.

Pharmacological compound	Animal/cell line	Experimental model	Main mechanism	Reference
A68930 (D1 dopamine receptor agonist)	Sprague-Dawley rats	Spinal cord injury	↓ NLRP3 ↓ IL-1, IL-18, TNF-α ↑ Locomotion recovery	Jiang et al. 2016 [229]
Adiponectin (Protein hormone)	Sprague-Dawley rats	Autologous blood model of intracerebral hemorrhage	↓ NLRP3, IL-1β, IL-18 ↓ Neurological deficits, ↓ Perihematomal brain edema	Wang et al. 2020 [230]
α1-antitrypsin (A1AT) (Protease inhibitor)	Primary cortical astrocyte cell culture (BALB/c mice)	Aβ_1-42_ and LPS stimulation	↓ NLRP3, caspase 1, IL-1β	Ebrahimi et al. 2018 [134]
Anfibatide (GPIb-IX-V complex antagonist)	Sprague-Dawley rats Primary cortical neuron cell culture (neonatal Sprague-Dawley rats)	Middle cerebral artery occlusion ischemia/reperfusion injury Oxygen-glucose deprivation and reintroduction	↓ Cerebral infarct volume ↓ Neurological deficits ↓ Bax, caspase-3 ↑ Bcl-2 ↓ NLRP3, ASC, caspase-1, IL-1β, IL-18 ↓ Neuronal apoptosis ↓ NLRP3, ASC, caspase-1, IL-1β, IL-18 ↓ NF-κB	Li et al. 2022 [231]
Atorvastatin (Inhibitor of hydroxymethylglutaryl-coenzyme A)	C57BL/6J mice	Surgery-induced BBB disruption	↑ Learning and memory ↓ NLRP3 ↓ IL-1, IL-6, TNF-α ↑ ZO-1, occludin, claudin 5	Liu et al. 2021 [232]
Caffeine (Antagonist of adenosine receptors)	BV2 microglial cell culture (C57BL/6J mice) Primary microglia cell culture (C57BL/6J mice) C57BL/6J mice	Experimental autoimmune encephalomyelitis	↓ mTOR ↓ Autophagy ↓ NLRP3 ↓ mTOR ↓ Autophagy ↓ NLRP3 ↓ Inflammatory cell infiltration, demyelination, microglial activation ↓ NLRP3 ↓ Autophagy	Wang et al. 2022 [233]
Calcitriol (1,25-dihydroxycholecalciferol./active form of vitamin D)	C57BL/6J mice	Experimental autoimmune encephalomyelitis	↓ ROS, NLRP3, ASC, caspase-1, IL-1β, ↓ CX3CR1, CCL17, RORc, GATA3, Foxp3, Tbx21 ↑ ZO-1	de Oliveira et al. 2020 [234]
Choline (quaternary ammonium cation, universal methyl donor)	APP/PS1 mice	Alzheimer’s disease model	↓ Aβ ↓ NLRP3 ↓ Microgliosis ↓ Cognitive deficits	Wang et al. 2019 [235]
CY-09 (Glitazone)	3xTg mice	Alzheimer’s disease model	↓ NLRP3, IL-1β, caspase-1 ↑ Glucose transport (GLUT1, GLUT4) ↓ Insuline resistance ↓ APP, Aβ, ROS ↑ PSD95, synaptophysin ↑ Cognitive function	Han et al. 2023 [236]
DAPANSUTRILE (OLT1177) 3-methylsulfonylpropanenitrile (NLRP3 inhibitor)	APP/PS1 mice Primary microglia cell culture (C57BL/6J mice)	Alzheimer’s disease model LPS stimulation	↑ Spatial memory ↑Spine density of hippocampal pyramidal neurons ↓ Microglia activation ↓ Aβ ↓ IL-1β, IL-6, TNF-α ↓ IL-1β, IL-6, TNF-α CLINICAL TRIAL NCT02104050, phase 2b, Moderate to severe pain associated knee osteoarthritis NCT03534297, phase 1b, Systolic heart failure and reduced ejection fraction	Lonnemann et al. 2020 [237]
Dihydromyricetin (Ampelopsin, (flavanonol)	APP/PS1 mice BV2 microglial cell culture (C57BL/6J mice)	Alzheimer’s disease model Aβ_1-42_ stimulation	↓ NLRP3, caspase-1, IL-1β ↑ Aβ clearance ↑ Cognitive function ↑ M2 microglial phenotype ↑ M2 microglial phenotype	Feng et al. 2018 [238]
Echinacoside (caffeic acid glycoside, phenylpropanoid)	Sprague-Dawley rats BV2 microglial cell culture (C57BL/6J mice)	Spinal cord injury LPS stimulation	↑Motor function recovery ↓Neuron loss ↓NLRP3, ASC, caspase-1, ↓ NF-κB, IL-1β, IL-18 ↓ NLRP3, ASC, caspase-1, ↓ NF-κB, IL-1β, IL-18 ↓ ROS	Gao et al. 2019 [239]
Fimasartan (Angiotensin II receptor antagonist)	Sprague-Dawley rats BV2 microglial cell culture (C57BL/6J mice)	Intracerebral hemorrhage Hemolysate treatment	↓Neurological functioning ↓Brain water content ↓Hematoma volume ↓ NLRP3, ASC, caspase-1, IL-1β ↓NF-κB ↓ NLRP3, ASC, caspase-1	Yang et al. 2018 [240]
Fluoxetin (serotonin reuptake inhibitor)	Sprague-Dawley rats	Social isolation induced depression AlCl3 induced Alzheimers’ disease	↑Improved motor performance ↑BDNF ↓ NF-κB, TLR-4, NLRP3, caspase-1, TNF-α, IL-1β ↓ AChE activity ↓Aβ, Tau ↓CK-MB, troponin, MEF2 ↑ Nrf2/HO-1	Abu-Elfotuh et al. 2022 [241]
Ghrelin (Protein hormon)	Sprague-Dawley rats	Experimental autoimmune encephalomyelitis	↓ Inflammatory brain infiltration, spinal cord demyelination ↓ TNF-α, IL-6, COX-2, iNOS ↓ Iba1, CD68 ↓ NLRP3, IL-1β, IL-18, ASC, caspase-1	Liu et al. 2019 [242]
Glibenclamide (sulfonylurea inhibitor)	Sprague-Dawley rats	2,5-hexanedione induced neurotoxicity	↓ NLRP3, caspase-1, pro-IL-1β, IL-1β, GSDM ↓ Oxidative stress, demyelination, axon degeneration ↓ Microglial M1 polarisation ↓ Iba1, CD11b	Hou et al. 2020 [243]
INZOMELID (IZD 174, MCC7840, Emlenoflast) 1-(1,2,3,5,6,7-hexahydro-s-indacen-4-yl)-3-(1-propan-2-ylpyrazol-3-yl) sulfonylurea			CLINICAL TRIAL NCT04015076, phase 1, Safety and Tolerability study in healthy and CAPS	
Ketamine (phencyclidine-derived dissociative anesthetic)	Wistar Kyoto rats Primary microglial cell culture (neonatal Sprague-Dawley rats)	Chronic restraint stress-induced depressive-like model LPS stimulation	↓ NLRP3, ASC, caspase-1, IL-1β ↑ BDNF, synaptophysin ↑ Autophagy	Lyu et al. 2022 [244]
KPT-8602 (inhibitor of exportin-1)	C57BL/6J mice Primary macrophage cell culture (C57BL/6J mice) Immortalised bone marrow-derived macrophages (C57BL/6J mice) BV2 microglial cell culture (C57BL/6J mice)	MPTP mouse model of Parkinson’s disease LPS stimulation + nigericin LPS stimulation LPS stimulation	↓ NLRP3, ASC, (pro)-caspase-1, (pro)-IL-1β ↓ IL-1β, IL-6, TNF-α ↓ Iba1 ↓NLRP3, ASC, caspase-1, IL-1β ↓NF-κB ↓IL-6, TNF-α, iNOS ↓IL-6, TNF-α, iNOS↓NLRP3 ↓NF-κB ↓NF-κB	Liu et al. 2022 [245]
Manoalide (calcium channel blocker)	C57BL/6J mice Bone marrow-derived macrophages / Peripheral blood mononuclear cells (C57BL/6J mice) THP-1 macrophage cell culture (human)	Experimental autoimmune encephalomyelitis LPS priming (canonical inflammasome) Nigericin, ATP or MSU (NLRP3 activation)Poly (dA:dT) transfection (AIM2 activation), Salmonella typhimurium infection (NLRC4 activation), C3 toxin (Pyrin activation) Pam3CSK4 (noncanonical inflammasome activation)	↓IL-1β, IL-6, TNF-α ↓NLRP3, ASC, (pro)caspase-1, (pro)-IL-1β, IL-18 AIM2, NLRC4, Pyrin are not inhibited by manoalide ↓NLRP3	Li et al. 2022 [246]
MCC950 (CP-456773, CRID3) 1,2,3,5,6,7-hexahydro-s-indacen-4-ylcarbamoyl-[4-(2-hydroxypropan-2-yl) furan-2-yl]sulfonylazanide	C57BL/6J mice	Perioperative neurocognitive disorder-model (explorative laparotomy)	↓ NLRP3, ASC, caspase-1, IL-1β, IL-18, TNF-α ↓ Iba1, GFAP ↑ BDNF, PSD95 CLINICAL TRIAL (ended) phase II against rheumatoid arthritis, discontinued because of liver toxicity	Fu et al. 2020 [247]
Milrinone (Inhibitor ofphosphodiesterase III	PP/PS1 mice BV2 microglial cell culture (C57BL/6J mice)	LPS/Aβ stimulation	↑ Memory function ↓ Aβ, p-Tau ↓ Iba1 ↓ Oxidative stress ↓ IL-1β, IL-6, TNF-α ↓NLRP3, ASC, caspase-1, IL-1β, IL-18 ↓ TLR4/MyD88/NF-κB	Chen et al. 2021 [248]
Minocycline (tetracycline antibiotic)	Wistar Kyoto rats	Toluene-induced memory impairment	↓ NLRP3, IL-1β, NF-κB ↓ CD11b, GFAP ↑ Memory function ↑ TGF-β ↓ ROS	Cruz et al. 2020 [249]
Mitoquinone(MitoQ), mitochondrial ROS antioxidant	C57BL/6J mice BV2 microglial cell culture (C57BL/6J mice)	Intracerebral hemorrhage-model FeCl_2_-treatment	↓ Brain edema, BBB leakage ↓ Neurological deficits (partially) ↑ M2 microglial polarisation) ↓ NLRP3, caspase-1, IL-1β, TNF-α ↑M2 microglial polarisation) ↓NLRP3, caspase-1 ↓ ROS	Chen et al. 2020 [250]
Nafamostat mesilate (wide-spectrum serine protease inhibitor)	Sprague-Dawley rats Primary microglial cell culture(postnatal Sprague-Dawley rats)	Middle cerebral artery occlusion Oxygen-glucose deprivation combined with thrombin	↓Infarct size ↑Improved behavioral functions ↓ TNF-α, IL-1β, iNOS, COX-2 ↑ CD206, TGF-β, IL-10, IL-4 ↓ NLRP3, NF-κB ↓ Infiltration of immune cells	Li et al. 2016 [251]
NT-0796 (isopropyl ester)			CLINICAL TRIAL NCT06129409, phase 1/2, obese participants with risk of cardiovascular disease (ongoing) ACTRN12621001082897 (safety, tolerability, pharmacokinetics and pharmacodynamics of NT-0796 in healthy volunteers)	
ORIDONIN (Diterpenoid, NSC-250682, Isodonol) 1-alpha,6-beta,7-alpha,14R)-7,20-Epoxy-1,6,7,14-tetrahydroxykaur-16-en-15-one	C57BL/6J mice	Traumatic brain injury	↓ NLRP3, ASC, caspase-1, IL-1β, IL-18 ↑ Claudin-5, occludin, ZO-1, caspase-3 ↑ Neurobehavioural performance ↓ Cerebral edema, cortical lesion volume, neuronal apoptosis CLINICAL TRIAL NCT05130892, Phase 4, Coronary artery disease (percutaneous coronary intervention)	Yan et al. 2020 [252]
Phoenixin-14 (Pleiotropic naturally occuring protein, GPR173 ligand)	Primary astrocyte cell culture (C57BL/6J mice)	LPS stimulation	↓ HMGB1- dependent NLRP3 activation↓ IL-1β, IL-18 ↑ eIF-2α, ATF4, CHOP ↓ ROS	Wang et al. 2020 [253]
Pramipexole (Dopamine D3 receptor agonist)	C57BL/6J mice Primary astrocyte cell culture (C57BL/6J mice or Drd3 knockout mice)	LPS injection model of Parkinson’s disease LPS and ATP stimulation	↓Loss of dopaminergic neurons ↓ NLRP3, ASC, (pro)-caspase-1, (pro)-IL-1β ↓ GFAP ↓ GFAP ↓ NLRP3, (pro)-caspase-1, (pro)-IL-1β ↑ Autophagy	Dong et al. 2023 [254]
Prednisone (corticosteroid, glucocorticoid receptor agonist)	C57BL/6J mice	Cuprizone-induced demyelination model	↑ Emotional behaviour, myelination ↓ Loss of weight ↓ GFAP, Iba1 ↓ NLRP3, ASC, caspase-1, IL-1β ↓ TNF-α, CCL8, CXCL10, CXCL16	Yu et al. 2018 [255]
Resolvin D1 (docosahexaenoic acid-derived lipid)	Sprague-Dawley rats	Subarachnoid hemorrhage	↓ NLRP3, NF-κB, MMP-9, ICAM-1 ↑ Occludin, claudin-5, ZO-1, A20	Wei et al. [256]
SELNOFLAST (SOMALIX, RO-7486967, IZD334) 1-(1-ethylpiperidin-4-yl)sulfonyl-3-(1,2,3,5,6,7-hexahydro-s-indacen-4-yl)urea			CLINICAL TRIAL NCT05924243, phase 1b, Parkinson’s disease (safety, tolerability, pharmacokinetics and pharmacodynamics of RO7486967 in participants with idiopathic PD)	
Sildenafil (3′,5′-cyclic GMP -specific phosphodiesteraseinhibitor)	APP/PS1 mice	Alzheimer’ disease model	↑ Memory function, locomotor activity ↑ cGMP/PKG/pCREB ↓ IL-1β, IL-6, TNF-α ↓ Aβ	Zhang et al. 2013 [257]
TRANILAST (RIZABEN) 2-{[(2*E*)-3-(3,4-Dimethoxyphenyl)prop-2-enoyl]amino}benzoic acid			CLINICAL TRIAL NCT05130892, Phase 4, Coronary artery disease (percutaneous coronary intervention) NCT01109121, phase 2, Hyperuricemia, gout NCT00882024, phase 2, Rheumatoid Arthritis	
1,2,4-Trimethoxybenzene	C57BL/6J mice Immortalised bone marrow-derived macrophages (C57BL/6J mice) Primary peritoneal macrophages (wild type and ASC KO mice) Primary mousemicroglia cell culture (C57BL/6J mice)	Experimental autoimmune encephalomyelitis LPS priming Nigericin/ATP induced NLRP3 activation	↓ Morbidity ↓ Loss of weight ↓ Demyelination ↓ NLRP3, ASC, caspase-1, IL-1β ↓ IFN-γ, IL-17a, CCL-5 ↑ IL-4 ↓ NLRP3, ASC, caspase-1, IL-1β ↓ NLRP3, ASC, caspase-1, IL-1β ↓ NLRP3, ASC, caspase-1, IL-1β	Pan et al. 2021 [258]
Urolithin A (gut bacteria transformed ellagitannins)	MPTP mice BV2 microglial cell culture (C57BL/6J mice)	Parkinson’s disease model LPS priming	↓ Motor deficits, dopaminergic neurodegeneration ↑ Mitophagy ↓ GFAP, Iba1 ↓ NLRP3, ASC, (pro)-caspase-1, (pro)-IL-1β ↓ ROS ↓ Mitochondrial dysfunction ↓ NLRP3, ASC, (pro)-caspase-1, (pro)-IL-1β ↓ TNF-α, iNOS, COX-2 ↓ ROS ↓ Mitochondrial dysfunction ↑ Mitophagy	Qiu et al. 2022 [259]
VTX2735			CLINICAL TRIAL NCT05812781, phase 1, CAPS	
VX-765 (Belnacasan, caspase-1 inhibitor)	APP/PS1 mice BV2 microglial cell culture (C57BL/6J mice)	Alzheimer’ disease model LPS priming	↓ NLRP3, ASC, (pro)-caspase-1, (pro)-IL-1β, IL-18, GSDMD, ↓ Aβ, p-Tau ↓CamKII, GSK-3β ↓Iba1 ↓ NLRP3, ASC, (pro)-caspase-1, (pro)-IL-1β, IL-18, GSDME ↓ AIM2 ↓CamKII, GSK-3β ↓ Aβ, p-Tau	Tian et al. 2022 [260]
ZYL1			CLINICAL TRIAL NCT05186051, phase 2a, CAPS NCT05981040, phase 2, Amyotrophic lateral sclerosis	

↓ decrease ↑ increase **Aβ:** Amyloid beta; **AchE:** Acetylcholinesterase; **APP/PS1:** Amyloid Precursor Protein-Presenilin 1; **ASC:** Apoptosis-associated speck-like protein containing a CARD; **ATF4:** Activating transcription factor 4; **ATP:** Adenosine triphosphate; **Bax:** Bcl-2-Associated X Protein*;* **BBB:** Blood-brain barrier; **Bcl2:** B-cell lymphoma 2; **BDNF**: Brain derived neurotrophic factor; **cAMP:** Cyclic adenosine monophosphate; **CCL:** CC chemokine ligand; **CamKII:** Ca^2+^Calmodulin-dependent protein kinase II; **CAPS:** Cryopyrin-Associated Periodic Syndromes; **CD:** Cluster of Differentiation; **cGMP/PKG/pCREB:** Cyclic guanosine monophosphate/protein kinase G/phosphorylated CREB; **CHOP:** C/EBP homologous protein; **CK-MB**: creatine kinase-MB; **CX3CR1:** C-X3-C Motif Chemokine Receptor 1; **eIF-2α:** Eukaryotic translation initiation factor 2A; **Foxp3**: Forkhead box P3; **GATA3**: GATA Binding Protein 3; **GFAP:** Glial Fibrillary Acidic Protein**; GSDM:** Gasdermin; **GSK3β:** Glycogen synthase kinase-3 beta; **HMGB1**: High mobility group box 1; **Iba1:** ionized calcium-binding adapter molecule 1; **ICAM-1:** Intercellular Adhesion Molecule 1; **IFN-γ**:Interferon gamma; **iNOS:** Inducible nitric oxide synthase*;* **IL:** Interleukin; **LPS:** Lipolysaccharide; **MDA:** Malondialdehyde; **MEF2:** Myocyte enhancer factor-2; **MYD88:** Myeloid differentiation primary response 88; **MMP-9:** Matrix metalloproteinase-9; **MPTP:**1-metil-4-fenil-1,2,3,6-tetrahidropiridin; **mTOR:** Mammalian target of rapamycin; **NF-κB:** Nuclear factor kappa-B**; NLRC:** NOD-like receptor family CARD-containing 4 protein; **NLRP:** Nod-like receptor protein; **NOX2**: NADPH oxidase 2; **Nrf2/HO-1:** Nuclear factor erythroid 2–related factor 2/HO-1: heme oxygenase-1; **PAR-1:** Proteinase-activated receptor 1; **PSD95:** Postsynaptic density 95; **RORc:** RAR-related orphan receptor C**; ROS:** Reactive oxygen species; **Tbx21:** T-box transcription factor 21; **TGF-β:** Transforming growth factor beta; **TNF-α**: Tumor necrosis factor alpha; **ZO-1:** Zonula Occludens-1

Further research by Saressella et al. [94] confirmed that both NLRP1 and NLRP3 inflammasomes may play a role in AD pathogenesis, with significant increases in the mRNA levels of various inflammasome components, including *NLRP1, NLRP3, PYCARD*, and *caspases 1, 5, and 8*, as well as downstream cytokines like *IL-1β* and *IL-18* in monocyte cultures derived from individuals with mild to severe cognitive decline.

#### 3.1.3 NLRP1 inflammasome in chronic neuropathic pain models

The involvement of NLRP1 in neuropathic pain was previously examined by Li et al. [95] utilizing the chronic constriction injury (CCI) model. Post-surgery, there was a notable increase in the levels of NLRP1, caspase-1, and ASC within neurons and glial cells, particularly in the superficial spinal dorsal horn. The observed increase in NLRP1 is correlated with elevated levels of IL-1β within the ipsilateral spinal cord, which significantly contributes to behaviors associated with neuropathic pain. It is also important to highlight that various neurodegenerative conditions such as AD, multiple sclerosis, spinal cord injuries, and traumatic brain injuries can all result in central neuropathic pain [96].

This dual involvement suggests that inhibiting NLRP1 may not only mitigate neurodegeneration in AD but also reduce chronic pain, underlining its potential as a therapeutic target for both conditions. To this point, the differences between species have greatly obstructed our understanding of the role of human NLRP1 in various diseases. Recently, a study led to the discovery of a new small-molecule dual inhibitor for NLRP1 and NLRP3, known as ADS032. This compound has been shown to decrease the release of IL-1β in macrophages and bronchial epithelial cells derived from humans [97]. Identifying additional effective inhibitors within the CNS for human NLRP1 or developing a "humanized" animal model would be instrumental in further exploring the function of NLRP1 in human neuroinflammatory disorders.

### 3.2 NLRP2 inflammasome

The NLRP2 (also known as NALP2, PYPAF2, PAN1) inflammasome was previously studied mainly because of its role in the reproductive system, as evidenced by its association with murine embryogenesis, age-related maternal fertility, and idiopathic recurrent miscarriage as one of the mammalian maternal effect genes [98-100]. Additionally, NLRP2 expression has been linked to arsenic-induced skin lesions, chromosomal damage, and respiratory disorders [101]. Minkiewicz et al. [102] were the first to reveal the functional role of NLRP2 as an inflammasome in human cortical astrocyte cultures, resolving its elusive status in the human CNS for many years.

#### 3.2.1 NLRP2 inflammasome in AD

Currently, there is limited data on the role of the NLRP2 inflammasome in neuropathological conditions [103, 104]. Recently, one study has elucidated the role of the NLRP2 in 5XFamilial Alzheimer’s disease (5XFAD) murine model of AD [105]. Glucagon-like peptide-1 (GLP-1) receptor agonist exenatide has been shown to diminish neuroinflammation in the piriform cortex, which subsequently enhances cognitive function in transgenic mice. Additionally, when cultured astrocytes were treated with exendin-4, a long-acting potent agonist of GLP-1, there was a notable reduction in levels of Aβ_1-42_, suggesting a decrease in inflammation and oxidative stress likely through the inhibition of NLRP2.

In our previous study, we utilized the STRING platform to map the human NLRP2 connectome. Our findings suggest that the NLRP2 network may play a crucial role in various neuronal processes, including glutamatergic excitotoxicity, apoptosis/survival signaling, neuroinflammation, and neurodegeneration [106].

**Table 4.** miRs Potentially Linked to Inflammasome Signaling in AD.

miRNA	Expression pattern in AD	Experimental model	Major signaling in disease	Reference
miR-101	downregulation	Postmortem human brain samples	↑COX-2	Nunez-Iglesias et al. 2010 [268]
Let-7	upregulation	C57BL/6J	↑TLR7	Lehmann et al. 2012 [269]
miR-34a	upregulation	CRL-2467 microglial cell culture (C3H/HeJ mice)	↓TREM2 ↑NF-κB	Zhao et al. 2013 [270]
miR-181	downregulation upregulation	Primary astrocyte cell culture (C57BL/6J mice) TNFR1/TNFR2 double knockout (C57BL/6J mice) 3xTg AD mice SH-SY5Y neuroblastoma cell culture (human)	↑HMGB1, TNFR, TLR4 ↑IL-1β, IL-8, IL-18 ↓IL-10 ↑c-Fos/SIRT1	Hutchison et al. 2013 [271] Rodriguez-Ortiz et al. 2014 [272]
miR-125b	upregulation	Human postmortem brain samples Primary cortical/hippocampal cell culture (Sprague- Dawley rats) C57/BL6 mice	↑p35, cdk5, p44/42-MAPK ↓Bcl-W, DUSP6, PPP1CA	Banzhaf-Strathmann et al. 2014 [273]
miR-155	upregulation	3xTg AD mice N9 microglia cell culture Primary astrocyte cell culture (C57BL6 mice)	↑IL-6, IFN-β ↑c-Jun/NF-κB ↓SOCS1 ↑Iba1, GFAP	Guedes et al. 2014; [274]
miR-126-3p	upregulation	Primary cortical/hippocampal cell culture (Sprague- Dawley rats/Tg6799 mice)	↓IGF-1/PI3K/AKT, ERK 1/2 ↓BDNF/trkB	Kim et al. 2016 [275]
miR-124–3p	downregulation	N2a/APP695swe neuroblastoma cell culture (mouse)	↑ Caveolin- PI3K/Akt/GSK3β	Kang et al. 2017 [276]
miR-21	downregulation	APP/PS1 mice Hypoxia-preconditioned mesenchymal stromal cells (C57BL6 mice)	↑STAT3 / NF-κB ↑Iba1, GFAP ↑IL-1β, TNF-α ↓IL-4, IL-10	Cui et al. 2018 [277]
miR-223-3p	downregulation	Serum samples from PD, AD, and MCI patients, and healthy controls	↑NLRP3	Mancuso et al. 2019 [278]
miR-29a	downregulation	Human peripheral blood mononuclear cells from AD patients	↑Wnt1/ ↑NF-κB ↓pCREB	Sedighi et al. 2019 [279]
miR-22	downregulation	Serum samples from AD, patients, and healthy controls APP/PS1 mice Primary microglia cell culture (mouse)	↑IL-1β, IL-18, TNF-α ↑NLRP3, caspase-1, GSDMD	Han et al. 2020 [280]
miR-132	downregulation	Sprague Dawley rats	↑MAPK1-iNOS	Deng et al. 2020 [281]
miR-9–5p	downregulation	Hippocampal neuron cell culture HT22 (mouse)	↑GSK-3β ↓Nrf2/Keap1	Liu et al. 2020 [282]
miR-16-5p	upregulation	5XFAD mice Primary cortical neuron cell culture ((C57BL6 mice) SH-SY5Y neuroblastoma cell culture (human)	↓BCL-2	Kim et al. 2020 [283]
miR-29c-3p	downregulation	STZ-induced AD model (C57BL6 mice) PC12 cells BM-MSC-EV cell culture (Sprague Dawley rats)	↑BACE1 ↑caspase-3,9, Bax ↑BACE1 ↓Wnt/β-catenin	Cao et al. 2021a [284] Sha et al. 2021 [285]
miR-590-3p	upregulation	*In silico* AD patients’ data	↑AMPK ↑NF-κB	Cao et al. 2021b [286]
miR-146a	upregulation	Serum samples from AD patients and healthy controls Hippocampal neuron cell culture (human)	↑NLRP3, caspase-1 ↑ IL-1β, IL-18 ↑NF-κB ↓p53/TIGAR ↑Oxidative stress	Lei et al. 2021 [287]
miR-483–5p	downregulation	Embryonic kidney 293 cell culture (human) Neuroblastoma SK-N-MC cell culture Neonatal dermal fibroblast cell culture (human)	↑ERK1/2/Tau	Nagaraj et al. 2021 [288]
miR-211	upregulation	PC12 cells	↓PI3K/AKT/Ngn2 ↑Caspase-3, Bax ↓BCL-2	Liu et al. 2021 [289]
miR-206-3p	downregulation	C57/BL6 mice	↓BDNF	Shao et al. 2022 [290]
miR-145-5p	upregulation	Plasma samples from AD patients and healthy controls	↓PI3K/AKT	Wen et al. 2024 [291]

↓ decrease ↑ increase **Aβ:** Amyloid beta; **AD:** Alzheimer’s disease; **AMPK:** 5′ AMP-activated protein kinase; **APP/PS1:** Amyloid Precursor Protein-Presenilin 1; **BACE:** Beta-Secretase; **Bax:**BCL-2 associated X protein; **BCL-2/W:** B-cell lymphoma 2/W; **BDNF:** Brain-derived neurotrophic factor*;* **BM-MSC-EV:** Bone marrow-mesenchymal stem cells -derived extracellular vesicles; **cdk5:** Cyclin-dependent kinase 5*;* **COX2:** Cyclooxygenase-2; **DUSP6:** Dual specificity phosphatase 6; **ERK1/2:** Extracellular signal-regulated kinase 1/2; **GSDMD:** Gasdermin *D*; **HMGB1:** High mobility group box 1; **IGF-1:** Insulin-like growth factor 1; **INF*γ***: Interferon-*γ*; **iNOS:** Inducible nitric oxide synthase*;* **MAPK:** Mitogen-activated protein kinase; **miR**: MicroRNA; **mTOR:** Mammalian target of rapamycin; **N/A:** Not applicable; **NF-κB:** Nuclear factor kappa-B; **Ngn2:** Neurogenin-2; **NLRP:** NOD-like receptor protein; **Nrf2/Keap1:** Nuclear factor erythroid 2-related factor 2/Kelch-like-ECH-associated protein 1; **pCREB:** phosphorylated cAMP response element-binding protein; **PI3K:** Phosphatidylinositol-3′-kinase; **PP2A:** Protein phosphatase 2A; **PPP1CA:** Protein phosphatase 1 catalytic subunit alpha; **SIRT1:** sirtuin-1; **SOCS1:** Suppressor of cytokine signaling 1; **STAT3**: Signal transducer and activator of transcription 3; **STZ:** Streptozotocin; TIGAR: TP53-induced glycolysis and apoptosis regulator; **TLR:** Toll-like receptor*;* **TNF-α**: Tumor necrosis factor-alpha; **TLR7:** Toll-like receptor 7; **TNFR:**Tumor necrosis factor receptor; **TREM2:**Triggering receptor expressed on myeloid cells 2; **trkB:** Tropomyosin receptor kinase B; **WNT:** Wingless-related integration site

#### 3.2.2 NLRP2 inflammasome in pain models

Matsuoka et al. [107] were among the first to investigate the role of NLRP2 expression in dorsal root ganglion (DRG) cells in the context of peripheral inflammatory pain induced by complete Freund’s adjuvant (CFA) or ceramide. Their findings demonstrated that silencing the NLRP2 gene in DRG cells using siRNA, as well as inhibiting caspase-1, effectively prevented nociceptive hypersensitivity.

In our study [108] using a CFA-induced rat pain model, we found that NLRP2 was primarily expressed in astrocytes of the spinal dorsal horn. While we observed a significant increase in NLRP2 protein levels in spinal cord tissue lysates, we did not determine its expression in DRG cells.

### 3.3 AIM2 inflammasome

Recently, the PYHIN family cytosolic DNA receptors have garnered increased attention due to their crucial roles in triggering innate immune responses. In humans, the family members consist of AIM2, IFN-γ inducible protein 16 (IFI16), interferon-inducible protein X (IFIX), and myeloid cell nuclear differentiation antigen (MNDA). Similarly, PYHIN family members have been identified in mice, such as AIM2, p202, p203, p204, and p205 [109]. AIM2 is a sensor protein that upon recognizing double-stranded DNA (dsDNA) undergoes oligomerization to combine with ASC and procaspase-1, forming a multiprotein hub, eventually leading to the secretion of bioactive proinflammatory cytokines (IL-1β, IL-18) and pyroptotic cell death.

Moreover, AIM2 inflammasome may trigger PANoptosis, a defense mechanism in the host characterized by the simultaneous activation of pyroptosis, apoptosis, and necroptosis [110]. Healthy neurons, microglia, and astrocytes in the mouse cerebral cortex and hippocampi express basal levels of the AIM2 protein [111,112]. Of note, *AIM2* deleted mice display decreased locomotor activity, heightened anxious behaviors and impaired auditory fear memory [113].

#### 3.3.1 AIM2 inflammasome in AD

Deletion of *AIM2* in APP/PS1 murine AD model was found to enhance dendrite branching and synaptic plasticity, leading to improvements in spatial memory. *AIM2* deletion in 5XFAD mice reduced Aβ deposition and microglial activation in the cortex and hippocampus [114]. Furthermore, microglia-specific *AIM2* deletion in Aβ_1-42_-induced AD model improved synaptic function and cognition. This was accompanied by reduced microglial activation and diminished synaptic phagocytosis, likely through inhibition of the classical complement pathway [115].

More recently, optineurin (OPTN) was identified as a regulator of neuroinflammation via suppression of AIM2 and RIPK1-mediated Nuclear factor kappa- B (NF-κB) signaling. In APP/PS1 transgenic mice, OPTN deficiency impaired mitophagy, leading to AIM2 inflammasome activation. Notably, OPTN overexpression counteracted Aβ-induced AIM2 activation by downregulating *AIM2* and *ASC* mRNA, reducing caspase-1 activation and IL-1β secretion from microglia [116].

Recent research has explored the role of extracellular vesicles in neurodegenerative diseases. Lark and LaRocca [117] demonstrated that alterations in gene expression related to multivesicular body and exosome formation are associated with AIM2 inflammasome activation in AD patients.

#### 3.3.2 AIM2 inflammasome in pain models

There is limited research available on the connection between AIM2 and chronic pain. Recently, Green-Fulgham et al. [118] has revealed that AIM2 signaling exhibits sex-specific differences in CCI-induced chronic neuropathic pain. Specifically, in female subjects, both NLRP3 and AIM2 are expressed at higher levels, whereas NLRP1 expression is more pronounced in males within the spinal lumbar cord following CCI.

### 3.4 NLRC4 inflammasome

NLRC4 is crucial for detecting Gram-negative bacteria in the cytoplasm and was initially termed ICE protease-activating factor (IPAF) for its role in caspase-1 activation [119, 120]. The NLRC4/IPAF inflammasome responds to bacterial flagellin and components of the bacterial type III (T3SS) and type IV (T4SS) secretion systems, detecting intracellular pathogens like *Salmonella typhimurium, Shigella flexneri, Pseudomonas aeruginosa*, and *Legionella pneumophila* [121]. Unlike other inflammasomes, NLRC4 can recruit procaspase-1 independently of ASC due to its CARD domain, though it requires interactions with NLR apoptosis inhibitory proteins (NAIPs) [122].

#### 3.4.1 NLRC4 inflammasome in AD

In neurodegeneration, Christie et al. [123] reported reduced NAIP-1 protein levels in the hippocampal and entorhinal cortices of AD patients. Subsequent studies linked NLRC4 activation to neuroinflammation. Ethanol exposure was shown to stimulate TLR4 signaling, leading to NLRC4/IPAF inflammasome activation in murine astrocytes and resulting in neuroinflammation and brain damage [124]. Similarly, palmitate-induced NLRC4 activation in astrocytes triggered IL-1β secretion, with ASC playing a crucial role in this process, as reducing NLRC4 or ASC levels significantly diminished IL-1β production [125]. Freeman et al. [126] further demonstrated that lysophosphatidyl-choline (LPC) activates both NLRC4 and NLRP3 inflammasomes via the G2A receptor (GPR132) in murine astrocytes and microglia.

Mejias et al. [127] found that proteins associated with the NLRC4 inflammasome, including ASC, caspase-1, and IL-18, were significantly elevated in the cerebral cortices of 18-month-old mice. Similarly, hippocampal lysates showed increased cytosolic levels of NLRC4, caspase-1, caspase-11, ASC, and IL-1β.

In a related study, Saadi et al. [87] observed significant upregulation of *NLRC4, ASC*, and *IL-1β* gene expression in Wistar rats following intracerebroventricular STZ administration.

#### 3.4.2 NLRC4 inflammasome in pain models

Limited information exists on the role of NLRC4 in pain conditions. However, its involvement was demonstrated in a carrageenan-induced acute inflammatory hyperalgesia model using *NLRP3, NLRC4*, and *ASC* knockout mice [128]. Behavioral assessments revealed that *NLRC4* and *ASC* knockout mice exhibited higher thresholds for mechanical and thermal pain responses. This reduced pain sensitivity correlated with lower levels of mature IL-1β and pro-caspase-1 at the inflammation site compared to wild-type mice.

### 3.5 NLRP3 inflammasome

NLRP3, the most extensively studied NLR family member, is activated by TLR agonists such as lipopolysaccharides (LPS) and inflammatory cytokines like TNF-α [129]. Beyond its role in immune defense, NLRP3 may also influence neurodevelopment and neurogenesis [130, 131], as its genetic deletion under normal conditions leads to reduced synaptic signaling, cognitive impairment, and anxiety-like behavior in 4-month-old mice [131].

NLRP3 expression is predominantly found in astrocytes and microglia within the CNS [63]. Interestingly, when Alois Alzheimer first documented AD in 1907, he noted abnormal glial cells surrounding Aβ plaques [132]. However, the activation of the NLRP3 inflammasome in astrocytes remains controversial. Gustin et al. [133] reported that mouse-derived astrocytes lacked NLRP3 expression, even after LPS exposure.

#### 3.5.1 Aβ -NLRP3 axis in astrocytes

Aβ accumulation is a key hallmark of AD, where glial activation contributes to neuronal damage. Ebrahimi et al. [134] reported that Aβ upregulates NLRP3 mRNA and protein in mouse astrocytes. Additionally, Aβ_1–42_ or LPS impairs autophagy and lysosomal function while activating the NLRP3/ASC/caspase-1/IL-1β pathway in primary mouse astrocytes and BV-2 cells under hypoxia. However, treatments with rapamycin, 17β-estradiol (E2), or progesterone restore autophagic activity and suppress NLRP3 inflammasome activation, whereas the autophagy inhibitor 3-methyladenine negates these protective effects and promotes NLRP3 activation [135, 136].

In a rat model, both oligomerized Aβ and H₂O₂ induced cellular senescence and enhanced IL-1β release from astrocytes via NLRP3 activation [137].

#### 3.5.2 Aβ -NLRP3 axis in microglia

Halle et al. [138] were the first to demonstrate that the phagocytosis of Aβ by microglia leads to the activation of the NLRP3 inflammasome. Since then, genetic methods such as GWAS have identified numerous genes specific to microglial cells that increase susceptibility to AD, including Triggering receptor expressed on myeloid cells 2 (TREM2), cluster of differentiation 33 (CD33), and complement receptor 1 (CR1) [139]. *In vitro* LPS stimulation of primary microglia corroborated TREM2 involvement in microglia-induced neuroinflammation [140].

Additionally, Wang et al. [141] demonstrated that both LPS stimulation and lentivirus-mediated over-expression of TREM2 significantly enhanced the activation of the NLRP3 inflammasome and promoted proinflammatory M1-type polarization of microglia in APP/PS1 mice. The release of IL-1β by microglial cells is promoted by Aβ through a mechanism that necessitates the production of reactive oxygen species (ROS) dependent on NADPH oxidase 2 (NOX-2) [142].

Recently, it has been shown that Aβ activated microglia triggers the NLRP3 inflammasome by recruiting Syk kinase and inhibiting AMPK in AD-like pathology (ADLP) mice. Inactive AMPK evokes metabolic dysregulation, mitochondrial fragmentation, generation of reactive oxygen species (ROS) [143]. NLRP3 activation by oligomeric Aβ also leads to a decrease in estrogen receptor α (ER-α) and the voltage-gated sodium-channel Na(v)1.1, which exacerbates neuroinflammation [144].

#### 3.5.3 Tau-NLRP3 axis

Phosphorylated Tau (p-Tau) is a key contributor to AD alongside Aβ. Stancu et al. [145] and Ising et al. [146] demonstrated that prion-like Tau seeds activate NLRP3 inflammasomes after being internalized by microglia and sorted into lysosomes in THY-Tau22 transgenic mice, a model of tauopathy. Jiang et al. [147] further showed that p-Tau exposure in human primary microglia upregulated *TLR2, TLR8, MyD88, p62, IRAK2*, and *IRAK3* mRNA expression. When microglia were exposed to neuronal media containing p-Tau/exosomes, IL-1β release was triggered via NLRP3/ASC/caspase-1-dependent mechanism.

Studies in rTg4510 and hTau mice revealed that reducing p-Tau or ASC levels decreased both Tau pathology and inflammasome activation. Consistently, Stancu et al. [148] reported that *Tau.NLRP3−/−* mice exhibited significantly attenuated Tau pathology in the hippocampus and cortex compared to *Tau.NLRP3+/+* mice. Additionally, NLRP3 deficiency mitigated prion-like Tau seeding and propagation in both ipsilateral and contralateral brain regions.

#### 3.5.4 NLRP3 related autophagy in AD

Autophagy plays a crucial role in microglial clearance of extracellular Aβ fibrils and in regulating Aβ-induced NLRP3 inflammasome activation. This has been demonstrated in both microglia-specific autophagy-related 7 *(Atg7)* knockout mice and *in vitro* models. Specifically, Aβ degradation is mediated through the MAP1-LC3B-II-OPTN axis, a pathway regulated by PRKAA1 (AMPK) signaling [149].

The role of Beclin-1 (BECN1) in autophagy is well established, particularly in coordinating autophagosome formation and cargo selection [150, 151]. Notably, microglia from *BECN1*+/- mice exhibit increased NLRP3 inflammasome activation, as evidenced by elevated levels of NLRP3, cleaved caspase-1, and the proinflammatory cytokines IL-1β and IL-18 [152]. In association with its binding partner, the class III phosphatidylinositol 3-kinase Vps34, BECN1 regulates intracellular trafficking by directing cellular components either to lysosomal degradation or back to the plasma membrane. A reduction in BECN1 impairs phagocytic receptor recycling, thereby compromising Aβ clearance.

Notably, postmortem analyses reveal a marked decline in BECN1 levels in microglia from AD patients, suggesting that dysregulated autophagy may contribute to both impaired Aβ clearance and sustained neuroinflammation in AD pathology [153].

Beyond microglia, studies have also highlighted the role of NLRP3 inhibition in promoting autophagy within astrocytes, suggesting a broader neuroprotective mechanism. Given the homeostatic functions of astrocytes, their autophagic regulation via inflammasome modulation may further influence Aβ clearance and neuroinflammatory responses in AD [135, 136].

#### 3.5.5 ER-NLRP3 axis in AD

During aging, the excessive accumulation of misfolded, unfolded, and aberrantly ubiquitinated or oxidized proteins in the endoplasmic reticulum (ER) contributes to proteostatic imbalances and structural abnormalities, which are hallmarks of neurodegenerative disorders. In AD, the buildup of Aβ and Tau proteins disrupts ER homeostasis, leading to progressive ER stress. In advanced stages, persistent ER stress becomes irreversible, ultimately triggering neuroinflammation and neuronal cell death [154].

Postmortem analyses of hippocampal tissues from AD patients reveal that ER stress activates the protein kinase RNA-like ER kinase (PERK) and inositol-requiring enzyme 1 (IRE1) pathways, leading to upregulation of thioredoxin-interacting protein (TXNIP). TXNIP antioxidant defenses while enhancing NLRP3 inflammasome activation, further driving neuro-inflammatory cascades. Additionally, elevated levels of key ER stress markers—including binding immunoglobulin protein (BiP), phosphorylated eukaryotic initiation factor-2α (eIF2α), and C/EBP homologous protein (CHOP)—underscore the critical role of ER dysfunction in AD pathology [155].

#### 3.5.6 GPCR-NLRP3 axis in AD

G-protein-coupled receptors (GPCRs) represent the largest receptor family comprising seven transmembrane domains that become activated upon binding their extracellular stimulus. Their conformational alterations trigger heterotrimeric G-proteins to initiate downstream signaling pathways by recruiting and activating cellular enzymes [156].

GPCRs may play a multifaceted role in neurodegenerative conditions, influencing disease progression and pathophysiology through NLRP3 inflammasome signaling [157]. Dopamine receptor dysregulation has long been recognized in AD, with postmortem studies revealing lower levels of dopamine D1 receptors in the putamen and hippocampus [158]. Since then, others has further detailed the expression patterns and alterations of dopamine receptors (D1–D5) in AD [159].

D1 and D2 receptors has been shown to mitigate Aβ-induced cognitive decline and neuroinflammation. Activation of the D1 receptor by A-68930 significantly attenuated NLRP3 inflammasome-mediated neuro-inflammation, likely via the AMPK/autophagy signaling pathway [160]. Similarly, bromocriptine, a D2 receptor agonist, promoted the recruitment of protein phosphatase 2A (PP2A) and c-Jun N-terminal kinase (JNK) by β-arrestin 2 in microglia, effectively suppressing proinflammatory cytokine transcription and NLRP3 activation [161].

Other GPCRs also modulate neuroinflammation in AD. For example, GPCR19 activation inhibits NLRP3-dependent inflammation by suppressing purinergic P2X7 receptor (P2X7R) signaling. Notably, taurodeoxycholate (TDCA), a GPCR19 ligand, blocked NLRP3 activation in 5XFAD mice while reducing P2X7R expression, Ca^2^⁺ mobilization, and IL-1β/IL-18 release by microglia. Additionally, TDCA enhanced Aβ phagocytosis and reduced Aβ plaques [162]. κ-opioid receptor agonist U50488H improved memory deficits in APP/PS1 mice by inhibiting the Ca^2^⁺/calmodulin-dependent protein kinase II/cAMP-response element binding protein (Ca^2^⁺/CaMKII/CREB) signaling pathway [163].

#### 3.5.7 Environment induced NLRP3 activation in AD

Environmental factors play a growing role in the progression and evolution of AD. Wang et al. [164] demonstrated that exposure to fine particulate matter (PM2.5) exacerbates oligomeric Aβ-induced neuronal damage, activates NLRP3 inflammasome, and increases ROS levels.

In addition, an increasing number of individuals are experiencing the adverse effects of arsenic exposure through contaminated drinking water. Arsenic and its byproducts contribute to oxidative stress, inflammation, mitochondrial dysfunction, ER stress, apoptosis, proteostasis disruption, and aberrant calcium signaling [165, 166]. Although research in this area remains limited, existing evidence suggests that arsenic exposure induces a hippocampal inflammatory response by upregulating the mRNA expression of proinflammatory cytokines *IL-6* and *TNF-α* via NLRP3 signaling, while simultaneously downregulating the anti-inflammatory cytokine *IL-10.* Furthermore, arsenic exposure reduces the mRNA levels of *T-helper 1 (Th1)* and *Th2* transcription factors, including *T-bet* and *GATA binding protein 3 (GATA3)*, as well as the cytokines *IFN-γ* and *IL-4*, suggesting broader immune dysregulation in AD [167].

#### 3.5.8 The gut-brain-NLRP3 axis in AD

In clinical settings, dietary interventions such as zinc supplementation have been shown to reduce the prevalence of AD and alleviate cognitive decline in APP/PS1 mouse models [168]. Beyond nutritional factors, the composition of the gut microbiota plays a crucial role in neurodegenerative conditions, influencing neuroinflammation through the microbiota-gut-brain axis [169].

A recent study utilizing the largest GWAS of gut microbiota genera from the MiBioGen consortium performed polygenic risk score (PRS) analysis using the "best-fit" model in PRSice-2. In a discovery cohort (ADc12 case/control: 1278/1293), researchers identified a genetic correlation between 119 bacterial genera and AD. A subsequent meta-analysis confirmed that ten genera were significantly associated with AD. Among these, four genera were linked to the apolipoprotein E (APOE) rs429358 risk allele in a manner consistent with their protective or risk-modifying effects. Notably, the proinflammatory genus *Collinsella*, identified as a risk factor for AD, exhibited a strong positive correlation with the APOE rs429358 risk allele in both sample sets [170].

Emerging evidence suggests that selenium nanoparticles coated with dihydromyricetin (DMY), chitosan, and a BBB-targeting peptide (Tg-CS/DMY@SeNPs) effectively inhibit Aβ aggregation and reduce NLRP3 activation in APP/PS1 mice. Additionally, these nanoparticles modulate inflammation-associated gut microbiota, including *Bifidobacterium, Dubosiella*, and *Desulfovibrio*, further underscoring the interplay between gut microbiota and AD pathology [171].

#### 3.5.9 NLRP3 activation in neuropathic pain

##### 3.5.9.1 NLRP3 activation in multiple sclerosis-associated neuropathic pain

A substantial body of evidence supports the involvement of NLRP3 inflammasome activation in the development and persistence of chronic neuropathic pain [172]. In experimental autoimmune encephalomyelitis (EAE) mouse model of multiple sclerosis (MS)-associated neuropathic pain, chronic oral administration of the NLRP3 inhibitor MCC950 progressively reversed neuropathic pain behaviors [173].

Additionally, MS-related neuropathic pain in myelin oligodendrocyte glycoprotein (MOG)-induced EAE mice is linked to complement system activation and NLRP3 inflammasome upregulation in lumbar DRG [174].

##### 3.5.9.2 NLRP3 activation in peripheral neuropathic pain

Chemotherapy-induced peripheral neuropathy (CIPN) is one of the most frequent and debilitating side effects of cancer treatment, surpassing bone marrow suppression and kidney dysfunction [175]. In a rat model of paclitaxel-induced neuropathic pain, NLRP3, caspase-1, and IL-1β expression were significantly elevated in CD68-labeled macrophages infiltrating L4–L6 DRG cells and the sciatic nerve. Paclitaxel also caused mitochondrial damage and ROS accumulation, triggering NLRP3 inflammasome activation [176].

Similarly, bortezomib treatment upregulated NLRP3 and signal transducer and activator of transcription 3 (STAT3) expression in DRG cells, while intrathecal administration of NLRP3 siRNA effectively prevented bortezomib-induced mechanical allodynia. Chromatin immunoprecipitation assays further revealed that bortezomib promoted STAT3 recruitment and increased histone H3 and H4 acetylation at the *NLRP3* promoter in DRG cells [177].

##### 3.5.9.3 NLRP3 activation in post-stroke pain

Ischemia/reperfusion injury following stroke is strongly associated with neuronal necrosis, apoptosis, and robust inflammatory response [178]. In a mouse model of central post-stroke pain, miR-223—an established negative regulator of the NLRP3 inflammasome—was significantly downregulated in the ipsilateral thalamus within one day of infarct induction. Notably, introducing a miR-223 antagomir into the ventral posterior lateral (VPL) nucleus of naïve mice replicated thalamic pain and elevated the expression of NLRP3, caspase-1, ASC, IL-1β, and IL-18 [179].

##### 3.5.9.4 NLRP3 activation in CCI

Other miRs, including miR-145, miR-223, miR-23a, miR-183, and miR-150, have also been implicated in neuropathic pain conditions such as CCI of the sciatic nerve [180–183]. For instance, miR-23a knockdown in naïve mice increased spinal TXNIP expression, inducing NLRP3 inflammasome activation, while miR-23a overexpression suppressed TXNIP/NLRP3 and alleviated neuropathic pain [183].

##### 3.5.9.5 NLRP3 activation in diabetic neuropathic pain

Diabetic neuropathy (DNP) is a severe and prevalent complication of diabetes mellitus, contributing to significant clinical burdens, including foot ulcers, neuropathic pain, and amputations [184].

In macrophage-derived monocytes from individuals with type 2 diabetes, exposure to DAMPs—such as ATP, high-mobility group protein B1, free fatty acids, islet amyloid polypeptide, and monosodium uric acid (MSU) crystals—triggered IL-1β and IL-18 maturation via mitochondrial ROS and NLRP3 inflammasome activation [185].

Consistently, findings from rat model of DNP also demonstrated increased ROS, NLRP3, TXNIP, caspase-1, IL-1β, and phosphorylated NMDA receptor subunit 2B (phospho-NR2B) in the lumbar spinal cord, further implicating NLRP3-mediated neuroinflammation in DNP pathogenesis [186].

##### 3.5.9.6 NLRP3 activation in complex regional pain syndrome

Complex regional pain syndrome (CRPS) is a debilitating condition characterized by sensory, autonomic, and trophic dysfunctions. Neurogenic inflammation plays a key role in symptom manifestation, particularly allodynia and hyperalgesia [187]. Conventional treatments such as nonsteroidal anti-inflammatory drugs (NSAIDs), corticosteroids, and opioids often fail to provide sufficient relief for CRPS-I (type I, without verified nerve injury), making its management challenging [188].

Spinal IL-1β and glial cell activation may contribute to the pain mechanisms of CRPS-I [189, 190]. Supporting this, intrathecal administration of the NLRP3-specific inhibitor MCC950 significantly reduced IL-1β levels and attenuated glial activation in the ipsilateral spinal dorsal horn in a rat chronic post-ischemic pain model of CRPS-I [191].

##### 3.5.9.7 NRLP3 activation in inflammatory pain models

In a CFA-induced murine pain model, proinflammatory markers—including NADPH oxidase 4 (NOX4), phosphorylated Janus kinase 2 (P-Jak2), phosphorylated signal transducer and activator of transcription 3 (P-Stat3), and NLRP3—were significantly upregulated in the lumbar spinal cord [192, 193].

Similarly, a study by Chen et al. demonstrated that intrathecal administration of the sphingosine-1-phosphate receptor 1 (S1PR1) agonist SEW2871 induced mechanical allodynia via NLRP3 inflammasome activation in the lumbar spinal dorsal horn [193]. Notably, the inhibition of S1PR1 with FTY720 prevented NLRP3 activation, while IL-10 blockade reversed the analgesic effects of FTY720, highlighting a potential regulatory interplay between S1PR1, NLRP3, and anti-inflammatory signaling pathways.

## 4. Contemporary strategies for managing chronic pain in dementia care

Corbett et al. [194] highlighted a critical gap in clinical practice: among fifteen pain management guidelines, only three address pain management in dementia patients. These guidelines predominantly emphasize pharmacological interventions, yet robust evidence supporting the long-term safety of commonly used analgesics is lacking.

Acetaminophen (paracetamol) remains the primary treatment for mild-to-moderate pain in dementia. Initially believed to act solely through cyclooxygenase (COX) inhibition, recent research now suggests that its mechanism involves modulation of spinal antinociceptive descending serotoninergic pathways [195]. Despite its widespread use, no studies have evaluated the efficacy and safety of paracetamol for treatment durations exceeding three months [196, 197].

NSAIDs, with their analgesic, anti-inflammatory, and antipyretic properties, are frequently prescribed for elderly patients. However, prolonged NSAID use is associated with significant adverse effects, including an increased risk of cardiac hypertension [198].

The use of opioid analgesics for dementia-related pain has grown substantially in recent decades. While opioids effectively manage severe pain, their use is fraught with challenges, including drowsiness, dizziness, increased fall risk, and fractures in elderly patients [199, 200]. Buprenorphine, for example, has been linked to adverse effects such as personality changes, confusion, and excessive drowsiness in nursing home residents with advanced dementia [201].

Tricyclic antidepressants (TCAs), including norepinephrine and serotonin uptake inhibitors, are occasionally used for pain management in dementia. However, their safety profile in older adults is concerned due to risks like the syndrome of inappropriate antidiuretic hormone secretion (SIADH) and hyponatremia [198].

Non-pharmacological interventions provide a promising alternative, with a recent systematic review [202] identifying interactive therapies such as singing, painting [203, 204], play-based activities [205], the therapeutic robot PARO [206], massage [207], ear acupressure [208], and music therapy [209].

## 5. Therapeutic targeting of NLRP3 in AD: clinical barriers

The global economic burden of AD and other dementias is rapidly escalating, with projections suggesting costs could soar to $16.9 trillion by 2050 unless groundbreaking advancements are achieved. This underscores the urgency of prioritizing AD research within public health initiatives [210].

Current pain management regimens for individuals with dementia are limited by significant side effects, making long-term treatment challenging. Cognitive decline is increasingly linked to chronic pain across various conditions, including fibromyalgia, postherpetic neuralgia, and chronic back pain [211].

While earlier research has emphasized the inhibition of inflammasomes such as NLRP1 (Table 1, [212-223]), NLRP2, AIM2, and NLRC4 (Table 2, [102, 105, 224-228]), preclinical and clinical studies have increasingly focused on targeting NLRP3 in neurological diseases (Table 3, [229-260]).

NLRP3 inflammasome plays a central role in neuroinflammation, making it an attractive therapeutic target. However, the effectiveness and safety of these treatments for AD remain largely unverified [63]. A major hurdle in inflammasome-targeted therapies is the substantial interspecies variation in genetic architecture, organ microenvironment, and cellular signaling between rodents and humans. These differences impact inflammasome activation and regulation, often leading to discrepancies between preclinical efficacy and clinical outcomes [261]. Furthermore, the precise mechanisms governing NLRP3 activation remain incompletely understood, particularly its interplay with nuclear receptors and the temporal regulation of IL-1β secretion. This gap in knowledge presents significant challenges for the rational design of selective inflammasome inhibitors [262, 263].

Further complicating clinical translation is the BBB, which restricts the penetration of therapeutic agents into the CNS. While FDA-approved IL-1β inhibitors such as anakinra, canakinumab, and rilonacept have shown promise in systemic inflammatory disorders, their limited ability to cross the BBB and the associated risk of opportunistic infections significantly constrain their utility. Therefore, for inflammasome-specific inhibitors to be viable in treating neuroinflammatory diseases, they must demonstrate clear advantages in terms of safety, efficacy, and BBB penetration [264-266].

## 6. miR-mediated crosstalk between neuro-inflammation, pain, and cognitive decline

In recent years, short non-coding RNA molecules, known as miRs, have gained attention for their regulatory roles in neurodegeneration. miRs undergo a tightly regulated biogenesis process, beginning with transcription by RNA polymerase II to generate primary miRs (pri-miRs). These are subsequently processed by the microprocessor complex (DROSHA-DGCR8) into precursor miRs (pre-miRs), which are exported to the cytoplasm via exportin 5 (XPO5). Once in the cytoplasm, Dicer further cleaves pre-miRs into mature miR duplexes, which are then incorporated into the RNA-induced silencing complex (RISC) for gene regulation [267].

**Figure 1.**
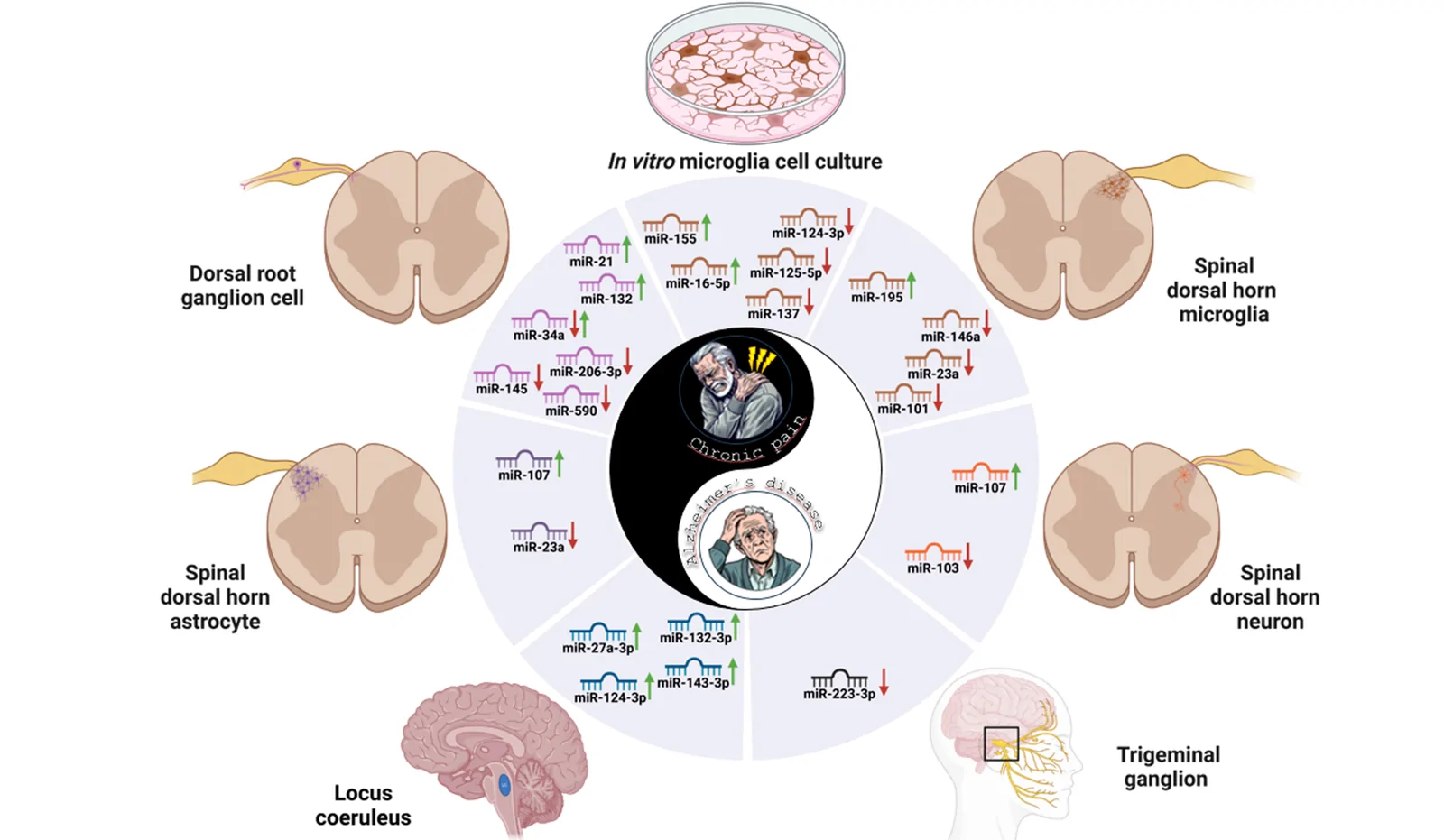
**Key miRNAs Implicated in Neuroinflammation During Alzheimer’s Disease and Chronic Pain, based on literature search "Created in BioRender**. Ducza, L. (2025) https://BioRender.com/o05z845". increase ↑ decrease ↓

While miRs are well-established as key regulators in neurodegenerative diseases such as AD [268], their potential role in linking neurodegeneration and chronic pain remains underexplored. Notably, several miRs associated with inflammasome activation or related signaling in AD (Table 4, [268–291]) may also contribute to chronic pain by modulating cellular processes in DRG and spinal dorsal horn, particularly in glial cells (Fig. 1).

The aforementioned LC-NE system may act as a central neuromodulatory hub. Under normal conditions, it suppresses nociceptive signaling, but in chronic pain, persistent hyperactivation leads to maladaptive neuroplasticity, intensifying pain sensitivity [292, 293]. The LC is one of the first regions affected in AD, showing nerodegeneration as early as Braak stage I–II. This early vulnerability is associated with dysregulated miR expression—notably miR-27a-3p, miR-124-3p, and miR-143-3p—which may disrupt LC-mediated pain inhibition [52]. These miRs, involved in neuroinflammation and synaptic plasticity, may thus form a molecular link between AD pathology and altered pain processing (Fig. 1).

Together, these findings point to a bidirectional neuroimmune loop: spinal neuroinflammation can accelerate AD progression, while neurodegeneration in AD can heighten spinal inflammation and pain sensitivity [28]. Clinically, this is evident as patients with advanced dementia often show increased pain sensitivity, and those with spinal cord injuries frequently develop cognitive and psychiatric complications [294].

## 7. Significance of the NLRP3 inflammasome–miR axis in AD and chronic pain

**Figure 2.**
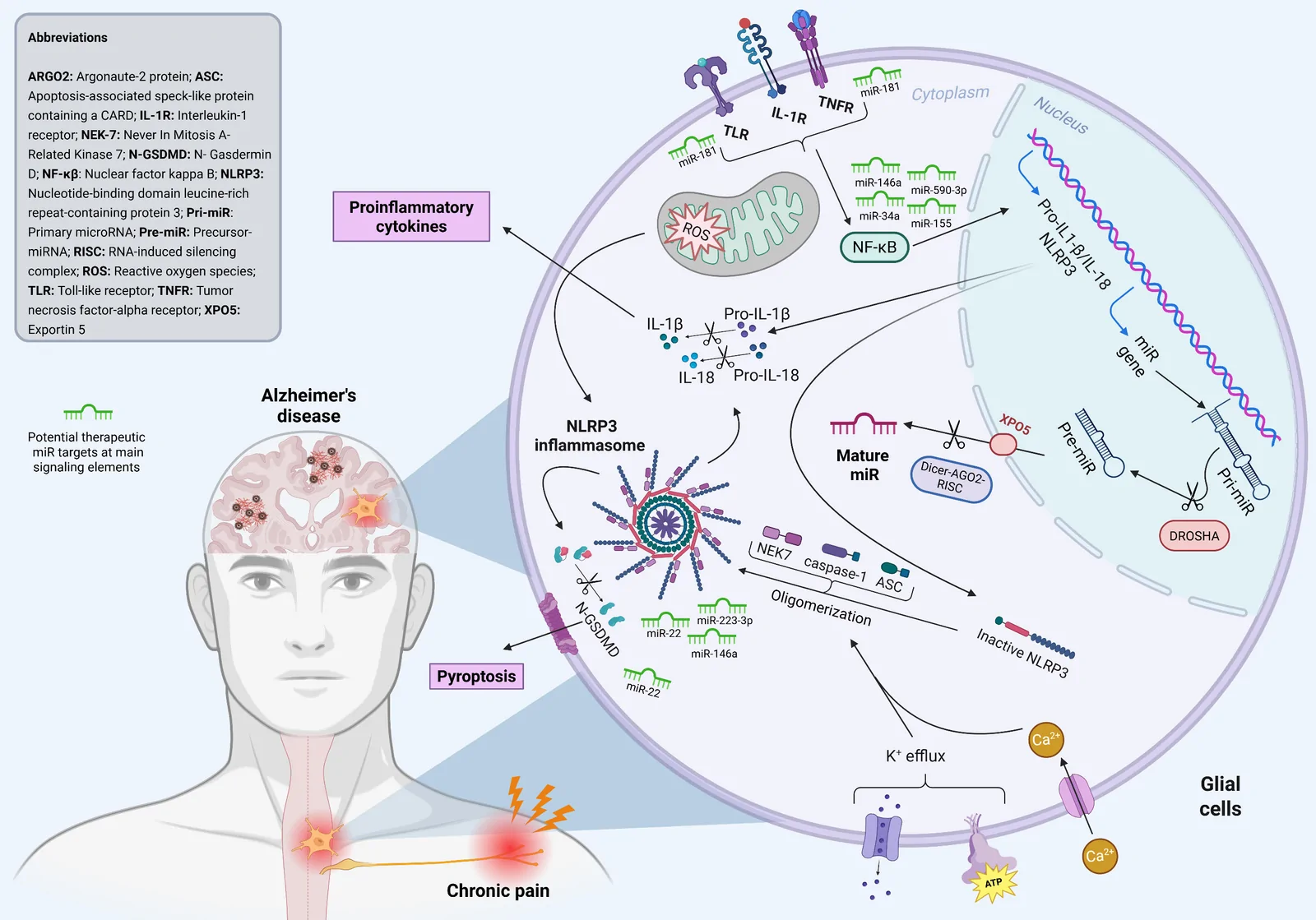
**The miR–NLRP3 Axis: A Molecular Bridge Between Neuroinflammation and Chronic Pain**, *Created in BioRender. Ducza, L. (2025) https://BioRender.com/o34t673*

While substantial research has delineated the roles of inflammasome activation and miR dysregulation in both AD and chronic pain, the therapeutic convergence of these pathways remains underexplored. NLRP3 inflammasome activation may act as a critical upstream modulator of neuroinflammatory signaling cascades across both conditions (Fig. 1 and Fig. 2).

Emerging evidence suggests that dysregulated miR expression in AD can amplify inflammatory responses, fueling NF-κB signaling (miR-34a, miR-590-3p, miR-146a, miR-155) [270, 274, 286, 287, 295, 296], even directly affects NLRP3 activation (miR-223-3p, miR-22, miR-146a) [278, 280, 287], while silencing key anti-inflammatory regulators such as suppressor of cytokine signaling 1 (SOCS1) (miR-155) and Sirtuin 1 (SIRT1) (miR-181) [277, 278, 296]. Furthermore, miR-22 and miR-181 may act as potent modulators of the proinflammatory signaling including IL-1R, TLR, and NLRP3 assembly, positioning them as critical nodes in the neuroimmune network [271, 272, 280] (Table 4).

Building on this molecular framework, miR-223 downregulation in post-stroke pain models was shown to elevate NLRP3-mediated IL-1β and IL-18 release, mimicking thalamic pain and exacerbating neuroinflammation [30, 179]. Thus, restoring miR-223 expression could provide analgesic and neuroprotective benefits.

Likewise, miR-34a is consistently implicated in neuropathic pain contexts, where its upregulation upon CCI enhances microglial activation and disrupts synaptic homeostasis by targeting SIRT1 and Vesicle-associated membrane protein 2 (VAMP2), ultimately contributing to central sensitization [297, 298]. miR-590-3p, downregulated in diabetic neuropathy, facilitates T cell recruitment through Ras-related protein 1A (RAP1A), amplifying nociceptive signaling in DRG [299].

miR-146a and miR-155, both central to NF-κB regulation, are robustly induced in pain models and act via targeting TNF receptor associated factor 6 (TRAF6) and SOCS1, respectively—modulating microglial and astrocytic reactivity, cytokine production, and mitogen-activated protein kinase (MAPK) activation [300, 301]. While miR-146a functions in a negative feedback loop to limit inflammatory signaling, sustained miR-155 expression promotes chronic glial activation and hyperalgesia.

## 8. Emerging multifaceted therapies centered on NLRP3 inhibition

Translating miR–inflammasome interactions from *in vitro* findings in animal and human cell cultures to *in vivo* studies in human glial cells could provide critical insights into neuroinflammatory disease mechanisms and open new avenues for targeted intervention. Notably, inconsistencies in miR expression patterns observed across AD studies [302] have complicated the identification of reliable therapeutic targets. Nevertheless, this regulatory axis holds promise as a future milestone in neuroimmune therapeutics, potentially enabling the development of precision medicine approaches tailored to individual inflammatory signatures.

Advances such as single-cell RNA sequencing have begun to resolve this complexity, revealing distinct microglial subpopulations with unique inflammasome-related gene signatures that may serve as cell-specific therapeutic targets [303]. In parallel, integrative tools like the STRING database [304] enhance our capacity to map the signaling networks linked to inflammasome activity, aiding in the identification and prioritization of regulatory nodes for targeted therapeutic intervention.

Recent advances in nanotechnology have introduced innovative strategies to enhance targeted brain delivery and therapeutic specificity in AD. Nanoparticle-based siRNA therapies administered via intracisternal injection have shown promise in achieving selective gene silencing within the CNS, thereby minimizing systemic side effects [305]. Similarly, guanidinium-modified calixarene/cyclodextrin nanocarrier systems have effectively delivered insulin across the blood–brain barrier, leading to improved cognitive function and reduced neuroinflammation in AD models [306]. Quercetin-functionalized nanomaterials also show considerable potential, particularly due to their capacity to inhibit NLRP3 inflammasome activation and attenuate neuroinflammation and oxidative stress, which are key contributors to AD pathology [307].

Naturally derived compounds also offer complementary therapeutic avenues. Apelin-13, for example, has demonstrated neuroprotective effects by modulating the BDNF–TrkB signaling pathway, suppressing neuroinflammation, and improving cognitive outcomes in AD models [308]. In parallel, traditional herbal formulations such as *Bushen Tiansu*i and *Lonicerae Japonicae Flos* exhibit anti-inflammatory and pro-cognitive effects, likely mediated through inflammasome-associated pathways such as NF-κB, MAPK signaling, among others [309, 310]. These botanicals contain a range of bioactive components, including flavonoids and phenolic acids, which may act synergistically to reduce oxidative stress, inhibit proinflammatory cytokine release, and protect neuronal integrity. Computational and systems biology approaches further suggest their involvement in modulating multiple AD-related targets, highlighting their potential as multi-modal therapeutics in neurodegenerative disease.

To further enhance translational relevance, emerging approaches such as the development of humanized animal models, human primary cell cultures, organ-on-chip systems, clustered regularly interspaced short palindromic repeats-CRISPR associated protein 9 (CRISPR-Cas9)–mediated gene editing are being employed to dissect species-specific inflammasome regulation [311]. Comparative omics and post-translational modification studies across species, along with strategies to overcome BBB limitations, are expected to yield deeper mechanistic insights into NLRP3 activation and inform the design of more selective, brain-penetrant inhibitors [312, 313].

Importantly, the complexity of neuroimmune crosstalk and its involvement in both cognitive and pain-related behaviors underscores the need for multi-targeted therapies. For example, models of anxiety, depression and endometriosis-induced pain have demonstrated that therapeutic modulation of glial activation and neuroinflammation leads to measurable behavioral and cognitive improvements [314, 315].

## Conclusions and Future Directions

Inflammasomes are key drivers of CNS neuroinflammation, contributing to chronic pain, Aβ accumulation, Tau hyperphosphorylation, synaptic dysfunction, and neurodegeneration. While their role in AD and chronic pain is well-documented, the direct connection between these conditions remains unclear, as much of the evidence comes from preclinical and observational studies rather than clinical trials.

miRs, as regulators of neurodegeneration and pain pathways, offer a promising yet underexplored therapeutic approach. Targeting the inflammasome-miR axis, particularly NLRP3 in glial cells, could provide novel strategies to alleviate chronic pain and slow AD progression. A precision medicine approach integrating molecular profiling may enhance treatment efficacy while minimizing adverse effects.

However, several challenges remain. Interspecies differences, inconsistent miR expression patterns, and limited clinical validation hinder therapeutic development. While advancements in bioinformatics and single-cell transcriptomics provide valuable insights, effective CNS-targeted delivery strategies are still lacking. Future research should prioritize validating these mechanisms in human tissues and refining inflammasome- and miR-based therapies to develop more precise interventions for neurodegenerative conditions and its comorbidities.

## Supplementary Materials

The Supplementary data can be found online at: www.aginganddisease.org/EN/10.14336/AD.2025.0353.
