# Supplementary material for: The Inflammasome–miR Axis in Alzheimer’s Disease and Chronic Pain: Molecular Mechanisms and Therapeutic Opportunities
Source: Aging Dis. 2025 May 21;17(3):1190–223. doi: 10.14336/AD.2025.0353 (PMC13061559; doi:10.14336/AD.2025.0353)
Supplement: Supplementary file 1 — The Supplementary data can be found online at: www.aginganddisease.org/EN/10.14336/AD.2025.0353. [file AD-17-3-1190-s.pdf]

SUPPLEMENTARY DATA

The Inflammasome–miR Axis in Alzheimer's Disease and Chronic Pain: Molecular Mechanisms and Therapeutic Opportunities

Botond Gaál, Roland Takács, Csaba Matta, Krisztián Juhász, Béla Fülesdi, Zoltán Szekanecz, Szilvia Benkő, László Ducza

© 2025. Gaál B et al. Published online at http://www.aginganddisease.org/EN/10.14336/AD.2025.0353

SUPPLEMENTARY DATA

Supplementary Table 1. NLRP3 Inflammasome Activation in Alzheimer's Disease

Axis / Cell Type	Mechanism of NLRP3 Activation	Pathological Effects in AD	Modulating Factors / Inhibitors	References
Astrocytes – Aβ axis	Aβ1–42, LPS impair autophagy → NLRP3/ASC/caspase-1/IL-1β pathway activation	Astrocyte senescence, increased IL-1β, Impaired clearance	Rapamycin, 17β-estradiol, progesterone inhibit NLRP3; 3-MA promotes it	[134–137]
Microglia – Aβ axis	Aβ phagocytosis → NLRP3 activation via ROS (NOX2), Syk kinase, AMPK inhibition	M1 polarization, IL-1β release, Mitochondrial dysfunction	TREM2 overexpression worsens; AMPK reactivation may inhibit it	[138–144]
Microglia – Tau axis	Prion-like p-Tau seeds → internalization → lysosomal stress → NLRP3 activation	IL-1β release, Inflammasome priming, Tau seeding/propagation	ASC knockout, NLRP3 knockout → reduced Tau pathology	[145–148]
Autophagy axis	Impaired MAP1-LC3B-II-OPTN/AMPK pathway and BECN1 function → NLRP3 activation	Impaired Aβ clearance, chronic inflammation	BECN1, AMPK promote autophagy and suppress NLRP3	[149–153]
ER stress axis	Misfolded proteins → PERK/IRE1 activation → TXNIP upregulation → NLRP3 activation	Neuroinflammation, neuronal death	TXNIP inhibition could attenuate NLRP3 activation	[154–155]
GPCR axis	D1/D2 dopamine receptor signaling modulates AMPK/autophagy/NLRP3;<br><br>P2X7R/GPR19 axis influences NLRP3	Cognitive improvement, less neuroinflammation with agonists	D1 agonist (A-68930), D2 agonist (Bromocriptine), TDCA (GPR19 agonist)	[156–163]
Environment (PM2.5, arsenic)	Oxidative stress, mitochondrial dysfunction, cytokine imbalance → NLRP3 activation	Increased IL-1β, IL-6, TNF-α; decreased IL-10, Th1/Th2 factors	Antioxidants, environmental control	[164–167]
Gut-Brain Axis	Aβ aggregation + gut microbiota dysbiosis → NLRP3 upregulation	IL-1β/IL-18 release, neuroinflammation	Selenium-DMY nanoparticles modulate microbiota + inhibit NLRP3	[168–171]

Abbreviations:
Aβ: Amyloid-β; **AD:** Alzheimer's disease; **AMPK:** 5' adenosine monophosphate-activated protein kinase; **ASC:** Apoptosis-associated speck-like protein containing a caspase recruitment domain; **BECN1:** Beclin1; **ER:** Endoplasmatic reticulum; **GPCR:** G-protein-coupled receptors; **IL:** Interleukin; **LPS: Lipopolysaccharide; MAP1-LC3B-II:** Microtubule-associated protein 1-light chain 3B protein; **NLRP: Nucleotide-binding domain leucine-rich repeat-containing protein; NOX2**: NADPH oxidase 2; **OPTN:** Optineurin; **PERK/IRE1:** Protein kinase receptor-like ER kinase/ inositol requiring enzyme 1; **P2X7:** P2X Purinergic receptor 7; **ROS:** Reactive oxygen species; **TDCA:** Taurodeoxycholic acid; **TXNIP:** Thioredoxin-interacting protein; **Th:** Helper T cell; **TNF-α**: Tumor necrosis factor-alpha; **TREM2:** Triggering receptor expressed on myeloid cells 2; **3MA:** 3-Methyladenine

© 2025. Gaál B et al. Published online at http://www.aginganddisease.org/EN/10.14336/AD.2025.0353

SUPPLEMENTARY DATA

Supplementary Table 2. NLRP3 Activation in Neuropathic Pain Conditions

Neuropathic Pain Type / Condition	Model or Source	Mechanism of NLRP3 Activation<br>Key Molecules/Pathways	Inhibition Strategy / Intervention	Reference(s)
MS-associated neuropathic pain	EAE mouse model	NLRP3 upregulation in lumbar DRG; complement activation<br><br>NLRP3, IL-1β, Caspase-1	MCC950 (oral)	[173, 174]
Chemotherapy-induced peripheral neuropathy (CIPN)	Sprague-Dawley rat model (paclitaxel, bortezomib)	Mitochondrial damage, ROS accumulation, STAT3 activation<br><br>NLRP3, Caspase-1, IL-1β, STAT3	NLRP3 siRNA, inhibition of STAT3 binding to promoter	[176, 177]
Post-stroke pain	CD1 mouse model (infarct, VPL microinjection)	Downregulation of miR-223 leads to NLRP3 activation<br><br>NLRP3, ASC, IL-1β, IL-18, Caspase-1	miR-223 mimic, miR-223 antagomir (induces pain)	[179]
Chronic constriction injury (CCI)	C57BL6 mouse model	miR-23a knockdown increases TXNIP expression<br><br>TXNIP, NLRP3	miR-23a overexpression	[183]
Diabetic neuropathic pain (DNP)	Human monocytes, Sprague-Dawley rat model rat model (streptozotocin)	DAMPs (ATP, HMGB1, etc.) trigger mitochondrial ROS<br><br>NLRP3, IL-1β, IL-18, Caspase-1, TXNIP, p-NR2B	Targeting ROS, NLRP3 pathway	[185, 186]
Complex regional pain syndrome (CRPS-I)	Sprague-Dawley rat model (chronic post-ischemic pain)	Glial activation, IL-1β increase via NLRP3<br><br>NLRP3, IL-1β	Intrathecal MCC950	[191]
Inflammatory pain	C57BL6 mouse model (CFA-induced)	NOX4, P-Jak2, P-Stat3 pathway activates NLRP3<br><br>NOX4, P-Jak2, P-Stat3, NLRP3	S1PR1 inhibition (FTY720), IL-10 modulation	[192, 193]

Abbreviations:
ASC: Apoptosis-associated speck-like protein containing a CARD; **ATP:** Adenosine triphosphate; **CD1:** Cluster of differentiation 1; **CFA:** Complete Freund adjuvant; **DAMP:** Damage-associated molecular pattern; **DRG:** Dorsal root ganglia; **EAE:** Experimental autoimmune encephalomyelitis; **HMGB1**: High mobility group box 1; **IL:** Interleukin; **JAK:** Janus kinase; **miR**: MicroRNA; **MS:** Multiple sclerosis; **NOX4**: NADPH oxidase 4; **NLRP3:** Nod-like receptor protein 3; **NR2B:** N-methyl D-aspartate receptor subtype 2B; **ROS:** Reactive oxygen species oxygen species; **STAT3:** Signal transducer and activator of transcription 3; **TXNIP:** Thioredoxin-interacting protein

© 2025. Gaál B et al. Published online at http://www.aginganddisease.org/EN/10.14336/AD.2025.0353
